# 2-Oxoester Phospholipase A_2_ Inhibitors with Enhanced Metabolic Stability

**DOI:** 10.3390/biom10030491

**Published:** 2020-03-24

**Authors:** Giorgos S. Koutoulogenis, Maroula G. Kokotou, Daiki Hayashi, Varnavas D. Mouchlis, Edward A. Dennis, George Kokotos

**Affiliations:** 1 Department of Chemistry, National and Kapodistrian University of Athens, Athens 15771, Greece; gkoutoul@chem.uoa.gr (G.S.K.); mkokotou@chem.uoa.gr (M.G.K.); 2 Department of Chemistry and Biochemistry and Department of Pharmacology, School of Medicine, University of California, San Diego, La Jolla, CA 92093-0601, USA; dhayashi@health.ucsd.edu (D.H.); vmouchlis@ucsd.edu (V.D.M.)

**Keywords:** inhibitor, metabolic stability, α-methylation, oxoesters, phospholipase A_2_

## Abstract

2-Oxoesters constitute an important class of potent and selective inhibitors of human cytosolic phospholipase A_2_ (GIVA cPLA_2_) combining an aromatic scaffold or a long aliphatic chain with a short aliphatic chain containing a free carboxylic acid. Although highly potent 2-oxoester inhibitors of GIVA cPLA_2_ have been developed, their rapid degradation in human plasma limits their pharmaceutical utility. In an effort to address this problem, we designed and synthesized two new 2-oxoesters introducing a methyl group either on the α-carbon to the oxoester functionality or on the carbon carrying the ester oxygen. We studied the in vitro plasma stability of both derivatives and their in vitro inhibitory activity on GIVA cPLA_2_. Both derivatives exhibited higher plasma stability in comparison with the unsubstituted compound and both derivatives inhibited GIVA cPLA_2_, however to different degrees. The 2-oxoester containing a methyl group on the α-carbon atom to the oxoester functionality exhibits enhancement of the metabolic stability and retains considerable inhibitory potency.

## 1. Introduction

Phospholipase A_2_ (PLA_2_) enzymes are involved in a variety of inflammatory diseases, which has stimulated the interest of the scientific community in exploring the pathophysiological role of each type of PLA_2_ and developing strategies for the modulation of their activity [1,2,3]. Among the various PLA_2_s present in mammals, the cytosolic calcium-dependent PLA_2_s (cPLA_2_) is characterized by a marked preference for the hydrolysis of arachidonic acid from the phospholipid substrates initiating the eicosanoid cascade [4]. The most well studied enzyme of this group is Group IVA (GIVA) cPLA_2_ and it is responsible for the biosynthesis of many diverse lipid signaling molecules contributing to inflammation [5]. Fundamental characteristics of the GIVA cPLA_2_, such as its role and function, have been summarized in recent review articles [6,7].

Early studies using gene targeted mice that lack GIVA cPLA_2_ have shown that these mice are much less prone to inflammatory pathological responses to disease, stresses and physical injuries, indicating the involvement of the enzyme in cellular and systemic damage [8,9]. The mechanisms regulating lipid peroxidation of arachidonic acid and docosahexaenoic acid in the central nervous system as well as the role of GIVA cPLA_2_ in oxidative and inflammatory signaling pathways in the central nervous system have been recently reviewed [10,11,12], highlighting the metabolic events linking this enzyme to activation in neurons, astrocytes, microglial cells, and cerebrovascular cells.

**Figure 1:**
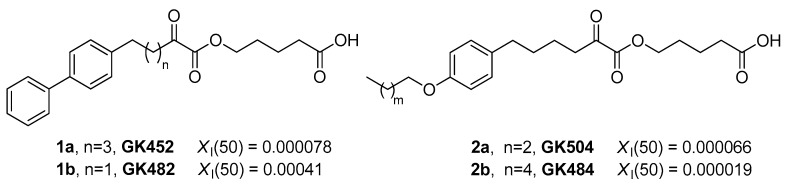
Known 2-oxoester inhibitors of GIVA cPLA_2_ [23,24].

A variety of small-molecule inhibitors have been developed as tools to understand the role of each type of PLA_2_ enzyme and as new medicinal agents [13,14]. Our groups have developed several classes of small-molecule PLA_2_ inhibitors [15,16,17,18,19,20] and have studied their in vitro inhibitory activities as well as their in vivo properties [21,22]. In particular, we have designed and synthesized a series of 2-oxoesters as a novel class of potent and selective inhibitors of human GIVA cPLA_2_ [23], having the ability to modulate the production of eicosanoids. The structural characteristics of these inhibitors are either a long fatty chain or an aromatic scaffold on the left, a 2-oxoester functionality and a short fatty chain with a terminal carboxylic group on the right (Figure 1). More potent 2-oxoester inhibitors were reported by our group [24], maintaining the same structural characteristics, but changing the substitution to an aromatic scaffold or altering the number of carbon atoms either to the left or to the right of oxoester group. Structure-activity relationship studies of these inhibitors allowed us to determine the necessary building blocks for optimizing inhibitory activity against GIVA cPLA_2_.

Among the many synthesized 2-oxoester GIVA cPLA_2_ inhibitors, four of them stood out as showing potent inhibition; their structures are shown in Figure 1. On the oxoester functionality (left side) of these inhibitors, there is an aliphatic chain containing two to four carbons bearing an aromatic scaffold, which is either a biphenyl system or a benzene ring with an aliphatic alkoxy group and on the ester functionality (right side) there is again a short aliphatic chain containing two to four carbons bearing a carboxyl group. As was shown, the four carbon atom chains on the left and on the right of the oxoester functionality are essential for the optimum inhibition of GIVA cPLA_2_, because when the number of carbon atoms on either side is decreased, the *X*_I_(50), defined elsewhere [25], increases, showing less potency [24].

Although the developed 2-oxoester inhibitors so far exhibited a potent in vitro inhibition of GIVA cPLA_2_, their rapid degradation in human plasma limits their pharmaceutical utility [24]. The aim of our work was to chemically modify the 2-oxoester inhibitors to increase their metabolic stability. We present herein the synthesis of methyl substituted 2-oxoesters, a study of their metabolic stability in plasma, and a study of their in vitro activity and specificity for three different PLA_2_s.

## 2. Materials and Methods

### 2.1. General Chemistry Methods

Chromatographic purification of products was accomplished using forced-flow chromatography on Merck^®^ (Merck, Darmstadt, Germany) Kieselgel 60 F_254_ 230–400 mesh. Thin-layer chromatography (TLC) was performed on aluminum backed silica plates (0.2 mm, 60 F_254_). Visualization of the developed chromatogram was performed by fluorescence quenching using phosphomolybdic acid, ninhydrin or potassium permanganate stains. Melting points were determined on a Buchi^®^ 530 (Buchi, Flawil, Switzerland) hot stage apparatus and are uncorrected. Mass spectra (ESI) were recorded on a Finningan^®^ Surveyor MSQ LC-MS spectrometer (Thermo, Darmstadt, Germany). High resolution mass spectrometry (HRMS) spectra were recorded on a Bruker^®^ Maxis Impact QTOF (Bruker Daltonics, Bremen, Germany) spectrometer. ^1^H and ^13^C NMR spectra were recorded on a Varian^®^ Mercury (Varian, Palo Alto, CA, USA) (200 MHz and 50 MHz, respectively), and are internally referenced to residual solvent signals. Data for ^1^H NMR are reported as follows: chemical shift (δ ppm), integration, multiplicity (s = singlet, d = doublet, t = triplet, m = multiplet, br s = broad signal), coupling constant and assignment. Data for ^13^C NMR are reported in terms of chemical shift (δ ppm).

*5-([1,1’-Biphenyl]-4-yl)-2-methylpentanoic acid* (**4**): Ιn a 25 mL round bottom flask containing a solution of diisopropylamine (1372 mg, 13.56 mmol) in extra dry tetrahydrofuran (THF) (6.0 mL) was added *n*-BuLi (8.47 mL, 1.6M, 13.56 mmol) under Ar, at 0 °C and the reaction mixture was left under stirring for 30 min. Next, a solution of compound **3** (1150 mg, 4.52 mmol) in extra dry THF (5 mL) was added and the resulting mixture was left stirring at 0 °C for 30 min. Hexamethylphosphoramide (HMPA) (2.20 mL) in extra dry THF (0.5 mL) was added and the resulting mixture continued stirring at 0 °C for 1 h. Finally, a solution of CH_3_I (905 mg, 5.96 mmol) in extra dry THF (1.5 mL) was added and the reaction mixture was left stirring at room temperature for 16 h. After completion of the reaction, a saturated aqueous solution of NH_4_Cl (15 mL) and Et_2_O (40 mL) were added. Then, hot water (3 × 15 mL) was added in the resulting mixture in order to dissolve the remaining HMPA. The aqueous layer was extracted by Et_2_O (3 × 15 mL) and the combined organic layers were dried over MgSO_4_. The solvent was removed under reduced pressure and the product was purified by column chromatography (petroleum ether 40–60 °C:EtOAc) (8:2). Yield 78%; Yellowish oil; ^1^H NMR (200 MHz, CDCl_3_): δ = 11.46 (br s, 1H), 7.67–7.29 (m, 9H), 2.73 (t, *J* = 7.1 Hz, 2H), 2.65–2.49 (m, 1H), 1.95–1.51 (m, 4H), 1.26 (d, *J* = 6.9 Hz, 3H); ^13^C NMR (50 MHz, CDCl_3_): δ = 183.8, 141.5, 141.4, 139.1, 129.1, 129.00, 127.4, 127.3, 39.6, 35.7, 33.4, 29.2, 17.2; HRMS [M-H^-^]: 267.1387; (calculated for [C_18_H_19_O_2_]^-^: 267.1391).

*5-([1,1’-Biphenyl]-4-yl)-2-methylpentan-1-ol* (**5**). To a solution of compound **4** (960 mg, 3.57 mmol) in dry THF (20 mL) was added ethyl chloroformate (0.51 mL, 5.35 mmol) followed by Et_3_N (0.75 mL, 5.35 mmol) at −10 °C and the reaction mixture was left stirring for 40 min. Next, NaBH_4_ (1080 mg, 28.56 mmol) was added and a subsequent dropwise addition of MeOH (25 mL) at 0 °C took place. The resulting reaction mixture was left stirring at room temperature for 16 h. The reaction mixture was evaporated under reduced pressure and was treated by HCl 1N until pH<7. The aqueous layer was extracted by EtOAc (3 × 20 mL) and the combined organic layers were washed by a 10% aqueous solution of NaHCO_3_ (20 mL). The organic layer was dried over MgSO_4_ and the solvent was removed under reduced pressure. The product was purified by column chromatography (petroleum ether 40–60 °C:EtOAc) (8:2). Yield 67%; Colourless oil; ^1^H NMR (200 MHz, CDCl_3_): δ = 7.68–7.29 (m, 9H), 3.62–3.42 (m, 2H), 2.71 (t, *J* = 7.5 Hz, 2H), 2.35 (br s, 1H), 1.85–1.52 (m, 4H), 1.35-1.19 (m, 1H), 1.00 (d, *J* = 6.6 Hz, 3H); ^13^C NMR (50 MHz, CDCl_3_): δ = 142.0, 141.4, 138.9, 129.1, 129.0, 127.3, 127.2, 68.4, 36.2, 35.9, 33.2, 29.12, 16.9; HRMS [M+Na]^+^: 277.1562; (calculated for [C_18_H_22_NaO]^+^ 277.1563).

*5-([1,1’-Biphenyl]-4-yl)-2-methylpentanal* (**6**). To a solution of compound **5** (600 mg, 2.36 mmol) in dry CH_2_Cl_2_ (25 mL), TEMPO (38.0 mg, 0.24 mmol) and iodobenzene diacetate (988 mg, 3.07 mmol) were added and the reaction mixture was left stirring at room temperature for 3 h. Next, a 10% aqueous solution of Na_2_S_2_O_3_ (10 mL) was added and the resulting reaction mixture was left stirring for 5 min. The organic layers were washed by H_2_O (20 mL) and dried over MgSO_4_. The solvent was removed under reduced pressure, the product was purified by column chromatography (petroleum ether 40–60 °C:EtOAc) (9:1), and was used directly for the next experiment. Yield 85%; Colourless oil.

*6-([1,1’-Biphenyl]-4-yl)-2-hydroxy-3-methylhexanenitrile* (**7**). To a solution of compound **6** (506 mg, 2.00 mmol) in CH_2_Cl_2_ (15 mL), a saturated aqueous solution of NaHSO_3_ (1.5 mL) was added and the reaction mixture was left stirring vigorously at room temperature for 30 min. The solvent was removed under reduced pressure and THF (15 mL) and H_2_O (10 mL) were added, followed by dropwise addition of a 4N aqueous solution of KCN (1.5 mL, 6.00 mmol). Then, the reaction mixture was left stirring at room temperature for 16 h. The reaction mixture was evaporated under reduced pressure and Et_2_O (30 mL) was added. The organic layer was washed with H_2_O (2 x 10 mL) and dried over MgSO_4_. The solvent was removed under reduced pressure and the product was purified by column chromatography (petroleum ether 40–60 °C:EtOAc) (8:2). Mixture of diastereomers; Yield 94%; Colourless oil; ^1^H NMR (200 MHz, CDCl_3_): δ = 7.65–7.20 (m, 9H), 4.36 (d, *J* = 5.5 Hz, 1H), 2.68 (t, *J* = 7.5 Hz, 2H), 2.61–2.43 (br s, 1H), 2.02–1.85 (m, 1H), 1.83–1.52 (m, 3*H*), 1.46–1.26 (m, 1H), 1.16–1.06 (m, 3H); ^13^C NMR (50 MHz, CDCl_3_): δ = 141.5, 141.4, 141.3, 139.1, 129.1, 129.0, 127.4, 127.3, 119.8, 119.5, 66.4, 66.0, 38.0, 35.8, 32.0, 31.5, 29.0, 28.9, 15.1, 14.9; HRMS [M+Na]^+^: 302.1511; (calculated for [C_19_H_21_NNaO]^+^ 302.1515).

*Methyl 6-([1,1’-biphenyl]-4-yl)-2-hydroxy-3-methylhexanoate* (**8**). A solution of compound **7** (525 mg, 1.88 mmol) in MeOH (6 mL) was treated with a freshly prepared 6N HCl in MeOH (6 mL) under stirring at room temperature for 16 h. The solvent was removed under reduced pressure and Et_2_O (30 mL) was added. The organic layer was washed with H_2_O (2 × 10 mL) and dried over MgSO_4_. The solvent was removed under reduced pressure and the product was purified by column chromatography (petroleum ether 40–60 °C:EtOAc) (8:2). Mixture of diastereomers; Yield 36%; White oil; ^1^H NMR (200 MHz, CDCl_3_): δ = 7.75–7.15 (m, 9H), 4.28–4.08 (m, 1H), 3.98–3.67 (m, 3H), 2.95 (br s, 1H), 2.82–2.55 (m, 2H), 2.18–1.90 (m, 1H), 1.81–1.58 (m, 2H), 1.49-1.21 (m, 2H), 1.18–0.77 (m, 3H); ^13^C NMR (50 MHz, CDCl_3_): δ = 176.0, 175.6, 141.9, 141.8, 141.3, 138.9, 129.1, 129.0, 127.3, 75.2, 73.5, 52.7, 52.6, 37.4, 37.0, 35.9, 35.8, 33.1, 30.7, 29.5, 29.2, 16.1, 13.8; HRMS [M+Na]^+^: 335.1613; (calculated for [C_20_H_24_NaO_3_]^+^ 335.1618).

*6-([1,1’-Biphenyl]-4-yl)-2-hydroxy-3-methylhexanoic acid* (**9**). To a solution of compound **8** (200 mg, 0.64 mmol) in MeOH (6 mL), a 1N aqueous solution of NaOH (5 mL) was added and the reaction mixture was left stirring at room temperature for 2 days. After acidification with 1N HCl (pH 1), the reaction mixture was extracted with Et_2_O (3 × 20 mL). The combined organic layers were washed with H_2_O (2 × 10 mL) and dried over MgSO_4_. The solvent was removed under reduced pressure and the product was purified by column chromatography (petroleum ether 40–60 °C:EtOAc) (8:2). Mixture of diastereomers; Yield 64%; White solid; mp: 147–151 ^o^C; ^1^H NMR (200 MHz, CD_3_OD): δ = 7.61–7.14 (m, 9H), 4.12–3.97 (m, 1H), 2.70–2.53 (m, 2H), 2.02–1.85 (m, 1H), 1.73–1.44 (m, 4H), 1.02–0.82 (m, 3H); ^13^C NMR (50 MHz, CD_3_OD): δ = 176.6, 176.2, 141.8, 141.2, 138.7, 128.8, 128.6, 126.8, 126.7, 126.6, 126.5, 74.5, 73.0, 37.0, 35.5, 35.4, 29.2, 15.4, 13.1; HRMS [M-H]^-^: 297.1492; (calculated for [C_19_H_21_O_3_]^-^ 297.1496).

*tert-Butyl 5-bromohexanoate* (**12**). In a 25 mL round bottom flask, δ-hexalactone (342 mg, 3.00 mmol) and 33% HBr in AcOH (5 mL) were added and the reaction mixture was left stirring at 75 °C for 16 h. After completion of the reaction, the reaction mixture was extracted with CH_2_Cl_2_ (3 × 15 mL). The organic layers were collected, washed with H_2_O (3 × 15 mL) and dried over MgSO_4_. The solvent was removed under reduced pressure and the formed bromo carboxylic acid was used directly to the next step. To a solution of the bromo carboxylic acid (544 mg, 2.82 mmol) in dry CH_2_Cl_2_ (20 mL) in a pressure vessel, concentrated H_2_SO_4_ (0.45 mL) was added dropwise. The reaction mixture was cooled at −196 °C using liquid nitrogen and isobutylene (20 mL) was added to the solution. The reaction mixture was left stirring at room temperature for 5 d. The reaction mixture was poured to a 100 mL beaker and was left stirring for 30 min. The reaction mixture was washed with H_2_O (3 × 15 mL) and the organic phase was dried over MgSO_4_. The solvent was removed under reduced pressure and the product was purified by column chromatography (petroleum ether 40–60 °C:EtOAc) (9:1). Yield 83%; Yellowish oil; ^1^H NMR (200 MHz, CDCl_3_): δ = 4.12–4.02 (m, 1H), 2.23-2.14 (m, 2H), 1.79–1.62 (m, 7H), 1.39 (s, 9H); ^13^C NMR (50 MHz, CDCl_3_): δ = 172.7, 80.4, 51.1, 40.4, 34.8, 28.3, 26.6, 23.4; HRMS [M+Na]^+^: 273.0467; (calculated for [C_10_H_19_BrNaO_2_]^+^ 273.0461).

General procedure for the synthesis of 2-hydroxy esters **15a,b**.

To a stirred solution of compound **9** or **13** (0.38 mmol) in THF (2.4 mL), a 20% aqueous solution of Cs_2_CO_3_ (124 mg, 0.38 mmol) was added (pH 9) and the reaction mixture was left stirring for 20 min at room temperature. The organic solvent was evaporated under reduced pressure and the residue was dissolved in DMF (6 mL). *tert*-Butyl ester **14** or **12** (0.46 mmol) was added and the reaction mixture was left stirring under reflux for 72 h to 168 h. Then, H_2_O (10 mL) was added and the reaction mixture was extracted with EtOAc (3 × 10 mL). The combined organic layers were dried over MgSO_4_ and the solvent was removed under reduced pressure. The product was purified by column chromatography (petroleum ether 40–60 °C:EtOAc) (8:2).

*5-(tert-Butoxy)-5-oxopentyl 6-([1,1’-biphenyl]-4-yl)-2-hydroxy-3-methylhexanoate* (**15a**). Mixture of diastereomers; Yield 35%; Colourless oil; ^1^H NMR (200 MHz, CDCl_3_): δ = 7.64–7.17 (m, 9H), 4.25–4.05 (m, 3H), 2.72-2.57 (m, 2H), 2.31–2.16 (m, 2H), 2.05-1.52 (m, 8H), 1.44 (s, 9H), 1.01 (d, *J* = 6.9 Hz, 1.5H), 0.84 (d, *J* = 6.9 Hz, 1.5H); ^13^C NMR (50 MHz, CDCl_3_): δ = 175.5, 175.1, 172.8, 141.9, 141.8, 141.3, 138.9, 134.1, 129.1, 129.0, 128.9, 127.2, 123.4, 80.6, 75.1, 73.4, 65.5, 65.4, 37.5, 36.9, 35.9, 35.1, 35.0, 33.1, 30.8, 29.6, 29.3, 28.3, 28.1, 21.7, 16.1, 13.7; HRMS [M+Na]^+^: 477.2608; (calculated for [C_28_H_38_NaO_5_]^+^ 477.2611).

*6-(tert-Butoxy)-6-oxohexan-2-yl 6-([1,1’-biphenyl]-4-yl)-2-hydroxyhexanoate* (**15b**). Mixture of diastereomers; Yield 37%; Colourless oil; ^1^H NMR (200 MHz, CDCl_3_): δ = 7.64–7.17 (m, 9H), 5.02–4.90 (m, 1H), 4.18–4.07 (m, 1H), 2.98–2.54 (m, 4H), 2.29–2.10 (m, 2H), 1.90–1.50 (m, 8H), 1.43 (s, 9H), 1.28-1.19 (m, 3H); ^13^C NMR (50 MHz, CDCl_3_): δ = 175.2, 172.8, 172.7, 141.8, 141.7, 141.3, 138.9, 129.0, 128.9, 127.3, 127.2, 80.5, 72.6, 70.7, 70.5, 35.7, 35.6, 35.2, 34.6, 34.5, 31.5, 31.4, 28.3, 24.8, 24.6, 21.0, 20.1, 20.0; HRMS [M+Na]^+^: 477.2614; (calculated for [C_28_H_38_NaO_5_]^+^ 477.2611).

General procedure for the synthesis of 2-oxoesters **16a,b**.

To a solution of compound **15** (0.13 mmol) in dry CH_2_Cl_2_ (2.0 mL) was added Dess-Martin periodinane (72 mg, 0.17 mmol) and the reaction mixture was left stirring at room temperature for 2 to 4 h. Then, a 10% aqueous solution of Na_2_S_2_O_3_ (5 mL) was added and the reaction mixture was extracted with CH_2_Cl_2_ (3 × 5 mL). The combined organic layers were mixed and dried over MgSO_4_ and the solvent was removed under reduced pressure. The product was purified by column chromatography (petroleum ether 40–60 °C:EtOAc) (8:2).

*5-(tert-Butoxy)-5-oxopentyl 6-([1,1’-biphenyl]-4-yl)-3-methyl-2-oxohexanoate* (**16a**). Yield 75%; Colourless oil; ^1^H NMR (200 MHz, CDCl_3_): δ = 7.63–7.19 (m, 9H) 4.24 (t, *J* = 6.3 Hz, 2H), 3.31–3.15 (m, 1H), 2.65 (t, *J* = 7.2 Hz, 2H), 2.25 (t, *J* = 6.9 Hz, 2H), 1.89-1.56 ( m, 8H), 1.44 (s, 9H), 1.15 (d, *J* = 7.0 Hz, 3H); ^13^C NMR (50 MHz, CDCl_3_): δ = 198.1, 172.7, 162.1, 141.2, 139.0, 129.0, 128.9, 127.3, 127.2, 127.1, 80.6, 66.0, 42.3, 35.6, 35.1, 31.7, 29.0, 28.3, 28.0, 21.6, 15.4; HRMS [M+Na]^+^: 475.2434; (calculated for [C_28_H_36_NaO_5_]^+^ 475.2455).

*6-(tert-Butoxy)-6-oxohexan-2-yl 6-([1,1’-biphenyl]-4-yl)-2-oxohexanoate* (**16b**). Yield 77%; Yellowish oil; ^1^H NMR (200 MHz, CDCl_3_): δ = 7.63–7.17 (m, 9H), 5.09–4.96 (m, 1H), 2.92–2.78 (m, 2H), 2.75–2.59 (m, 2H) 2.29–2.12 (m, 2H), 1.83–1.52 (m, 8H), 1.48–1.22 (m,12H); ^13^C NMR (50 MHz, CDCl_3_): δ = 194.9, 172.7, 161.1, 141.3, 139.0, 129.0, 128.9, 127.3, 127.2, 80.5, 73.7, 39.4, 35.4, 35.2, 35.1, 30.9, 28.3, 22.8, 21.0, 19.9; HRMS [M+Na]^+^: 475.2456; (calculated for [C_28_H_36_NaO_5_]^+^ 475.2455).

General procedure for the synthesis of 2-oxoesters **17a,b**.

A solution of *tert*-butyl ester **16** (0.13 mmol) in dry CH_2_Cl_2_ (3.0 mL) and TFA (3.0 mL) was stirred at room temperature for 2 h. The solvent was removed under reduced pressure and then CH_2_Cl_2_ (5 mL) was added and re-evaporated twice. The product was purified by column chromatography (petroleum ether 40–60 °C:EtOAc) (8:2).

*5-((6-([1,1’-Biphenyl]-4-yl)-3-methyl-2-oxohexanoyl)oxy)pentanoic acid* (**17a**) (GK587). Yield 77%; Colourless oil; ^1^H NMR (200 MHz, CDCl_3_): δ = 7.63–7.16 (m, 9H), 4.25 (t, *J* = 6.3 Hz, 2H), 3.34–3.12 ( m, 1H), 2.65 (t, *J* = 7.2 Hz, 2H), 2.39 (t, *J* = 6.8 Hz, 2H),1.91–1.54 (m, 8H), 1.15 (d, *J* = 7.0 Hz, 3H); ^13^C NMR (50 MHz, CDCl_3_): δ = 198.0, 179.4, 162.0, 141.2, 139.0, 129.0, 128.9, 127.3, 127.2, 65.9, 42.3, 35.6, 33.5, 31.6, 29.0, 27.9, 21.2, 15.4; HRMS [M-H]^-^: 395.1854; (calculated for [C_24_H_27_O_5_]^-^ 395.1864).

*5-((6-([1,1’-Biphenyl]-4-yl)-2-oxohexanoyl)oxy)hexanoic acid* (**17b**) (GK639). Yield 87%; Colourless oil; ^1^H NMR (200 MHz, CDCl_3_): δ = 7.62-7.17 (m, 9H), 5.10–4.94 (m, 1H), 2.94–2.77 (m, 2H), 2.73–2.57 (m, 2H), 2.44-2.28 (m, 2H),1.85–1.52 (m, 8H), 1.30 (d, *J* = 7.0 Hz, 3H); ^13^C NMR (50 MHz, CDCl_3_): δ = 194.9, 179.5, 161.1, 141.3, 139.0, 129.0, 128.9, 127.3, 127.3, 127.2, 73.6, 39.4, 35.4, 35.0, 33.7, 30.9, 22.8, 20.6, 19.9; HRMS [M-H]^-^: 395.1857; (calculated for [C_24_H_27_O_5_]^-^ 395.1864).

### 2.2. Plasma Stability Studies

For plasma stability, LC-MS studies were performed with an ABSciex Triple TOF 4600 (ABSciex, Darmstadt, Germany) combined with a micro-LC Eksigent (Eksigent, Darmstadt, Germany) and an autosampler set at 5 °C and a thermostated column compartment. Electrospray ionization (ESI) in negative mode was used for the MS experiments. The data acquisition method consisted of a TOF-MS full scan *m/z* 50–850 Da and several IDA-TOF-MS/MS (Information Dependent Acquisition) product ion scans using 40 V Collision Energy (CE) with 15 V (Collision Energy Spread) CES used for each candidate ion in each data acquisition cycle (1091). Halo C18 2.7 μm, 90 Å, 0.5 × 50 mm^2^ from Eksigent was used as a column and the mobile phase consisted of a gradient (A: acetonitrile/0.01% formic acid/isopropanol 80/20 v/v; B: H_2_O/0.01% formic acid). The elution gradient adopted started with 5% of phase B for 0.5 min, gradually increasing to 98% in the next 7.5 min. These conditions were kept constant for 0.5 min, and then the initial conditions (95% solvent B, 5% solvent A) were restored within 0.1 min to re-equilibrate the column for 1.5 min for the next injection (flow rate 55 µL/min). The data acquisition was carried out with MultiQuant 3.0.2 and PeakView 2.1 from AB SCIEX (ABSciex, Darmstadt, Germany). 

### 2.3. In vitro PLA_2_ Activity Assay

Group specific PLA_2_ assays were employed to determine the inhibitory activity using a lipidomics-based mixed micelle assay as previously described [4,25]. The substrate for each enzyme consisted of 100 µM PAPC (except for GIVA cPLA_2_ as noted), 400 µM of C12E8 surfactant, and 2.5 µM of 17:0 LPC internal standard. For GIVA cPLA_2_, the total phospholipid concentration (100 µM) consisted of 97 µM PAPC and 3 µM of PI(4,5)P_2_ which enhances the activity of the enzyme. A specific buffer was prepared to achieve optimum activity for each enzyme. The buffer for GIVA cPLA_2_ contained 100 mM HEPES pH 7.5, 90 µM CaCl_2_, and 2 mM DTT. For GVIA iPLA_2_, the buffer consisted of 100 mM HEPES pH 7.5, 2 mM ATP, and 4 mM DTT. Finally, the buffer for GV sPLA_2_ contained 50 mM Tris-HCl pH 8.0 and 5 mM CaCl_2_. The enzymatic reaction was performed in a 96 well-plate using a Benchmark Scientific H5000-H MultiTherm heating shaker for 30 min at 40 °C. Each reaction was quenched with 120 µL of methanol/acetonitrile (80/20, v/v), and the samples were analyzed using a HPLC-MS system. A Shimadzu SCL-10A system controller with two LC-10AD liquid pumps (Shimadzu Corp.,Kyoto Kyoto, Japan) connected to a column controller instrument (Analytical Sales & Products, Inc, Flanders NJ, USA), and a CTC Analytics PAL autosampler platform (Leap Technologies, NC, USA) were used for HPLC analysis. An AB Sciex 4000 QTRAP triple quadrupole/linear ion trap hybrid mass spectrometer (AB Sciex LLC, Framingham MA, USA) was used for MS analysis. A blank experiment, which did not contain enzyme, was also included for each substrate to determine the non-enzymatic hydrolysis product and to detect any changes in the intensity of the 17:0 LPC internal standard.

## 3. Results and Discussion

### 3.1. Design and Synthesis of Inhibitors

We assumed that in human plasma, it is likely that the oxoester functionality is hydrolyzed by an esterase. We hypothesized that if a small organic functionality, such as a methyl group, was added, either on the α-carbon to the oxoester functionality or on the α-carbon to the ester oxygen, it would decrease the rate of destruction of these compounds. The methyl group is the smallest alkyl group and has played a beneficial role in drug design by often constituting the problem-solving key to lead optimization [26]. Mainly, the PharmacoKinetic (PK) and PharmacoDynamic (PD) properties of a compound can be modified by the addition of the methyl group, where in many cases an increase in the selectivity and potency of the pharmaceutical agent has resulted.

**Figure 2:**
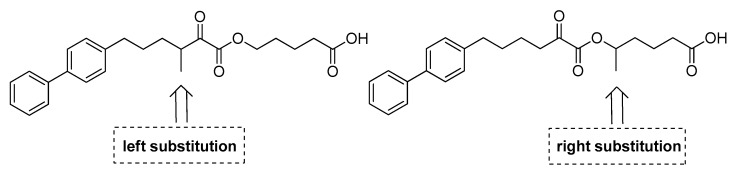
Design of α-methyl derivatives of **GK452** inhibitor of GIVA cPLA_2_.

GK452 (**1a**, Figure 1) was one of the most potent 2-oxoester inhibitors of GIVA cPLA_2_. Thus, we envisaged that the two methylated derivatives of this compound shown in Figure 2 should result in an increased metabolic stability. 

The synthetic strategy for how to insert the methyl group in the desired positions (left or right of the oxoester) was, firstly, with a lithium diisopropylamide (LDA) treatment followed by methylation for the left addition, and by a coupling reaction of an intermediate α-hydroxy carboxylic acid to a methylated secondary bromo *tert*-butyl ester for the right addition. 

**Scheme 1:**
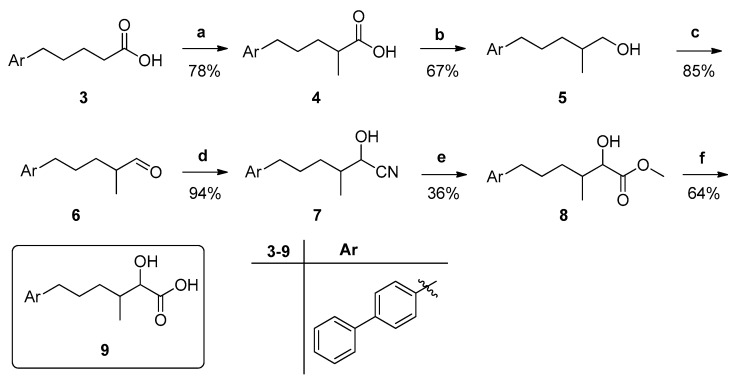
Synthesis of 2-hydroxy acid **9**. (**a**) (i) LDA, dry THF (ii) HMPA, (iii) CH_3_I; (**b**) (i) CH_3_CH_2_OCOCl, Et_3_N, dry THF, (ii) NaBH_4_, MeOH; (**c**) iodobenzene diacetate, TEMPO (10 mol%), dry CH_2_Cl_2_; (**d**) aq. sol. NaHSO_3_, CH_2_Cl_2_, (ii) KCN, H_2_O; (**e**) 6N HCl/MeOH; (**f**) 1N NaOH, MeOH.

The synthesis of the needed α-hydroxy β-methyl carboxylic acid **9**, bearing a biphenyl scaffold on the left, is described in Scheme 1. The first reaction was an α-substitution using an LDA method, hexamethylphosphoramide (HMPA) to help stabilize the formed enolate and CH_3_I as the electrophile [27], giving the α-methyl carboxylic acid **4** in a high yield. Then, the carboxylic moiety was reduced to an alcohol by converting it into the corresponding mixed anhydride and finally reducing it with NaBH_4_ in the presence of MeOH [28]. Alcohol **5**, which was obtained in a high yield, was next oxidized using PCC to aldehyde **6**, which after reaction with KCN gave cyanohydrin **7** in an excellent yield. Acidic methanolysis by treatment with freshly prepared 6N HCl in MeOH led to the corresponding hydroxy methyl ester **8** in a good yield, which finally, after saponification, afforded the desired α-hydroxy carboxylic acid **9**. 

**Scheme 2:**
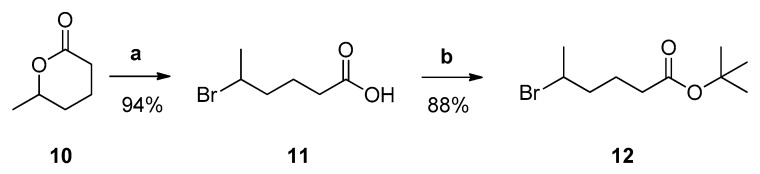
Synthesis of bromo *tert*-butyl ester **12**. (**a**) 33% HBr in AcOH; (**b**) isobutylene, c.H_2_SO_4_, dry CH_2_Cl_2_.

For the synthesis of *tert*-butyl ester **12**, acidic bromination of commercially available delta-hexalactone **10**, using 33% HBr in AcOH [29], produced the intermediate bromo carboxylic acid **11** in almost quantitative yield. Next step was the protection of the carboxyl group using isobutylene in the presence of c. H_2_SO_4_, leading to *tert*-butyl ester **12** in a high yield (Scheme 2).

**Scheme 3:**
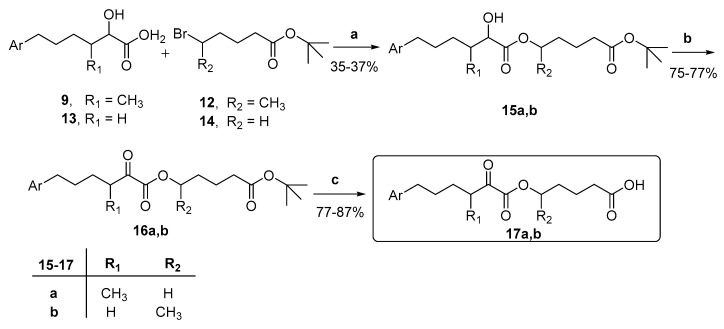
Synthesis of 2-oxoesters. (**a**) (i) Cs_2_CO_3_, THF, H_2_O (pH 9); (ii) DMF, reflux, 24–72 h; (**b**) Dess Martin, dry CH_2_Cl_2_, r.t., 3 h; (**c**) 50% TFA, dry CH_2_Cl_2_, r.t., 2 h.

After having synthesized the needed acid **9** and the appropriate hydroxy acid **13**, as previously described [23], a coupling reaction was performed by treatment with Cs_2_CO_3_ and the corresponding bromo *tert*-butyl esters (methylated or not) (Scheme 3). The coupling products **15a** and **15b** were produced in moderate yields 35–37%. For this step, it was observed that, when *tert*-butyl ester **12** was used, an increased reaction time was needed. Oxidation using Dess-Martin periodinane in dry CH_2_Cl_2_ gave oxoesters **16a** and **16b** in high yields. Final deprotection of the *tert*-butyl ester by using 50% TFA in dry CH_2_Cl_2_ led to the desired products **17a** (GK587) and **17b** (GK639) methylated derivatives.

### 3.2. Plasma Stability Studies

**Figure 3:**
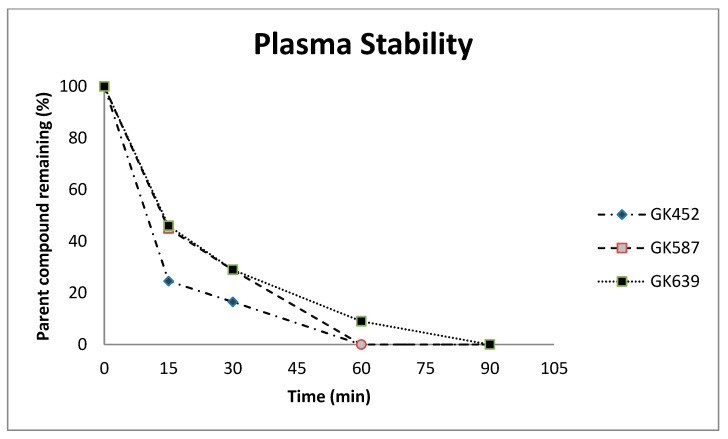
Plasma stability of GK587 and GK639 in comparison with GK452.

The in vitro stability of 2-oxoesters **17a** and **17b**, as well as inhibitor GK452 for comparison, was studied in human plasma. The reactions were initiated by the addition of test compound to a preheated plasma solution to yield a final concentration of 1 mg/L [24]. Samples (50 μL) were obtained and after the appropriate treatment were analyzed by LC-HRMS/MS (ABSciex, Darmstadt, Germany) and the results are shown in Figure 3 (see, also Supplementary Material).

An approximately 85% increase on the metabolic stability was observed at 15 min for both compounds; the parent compound remaining was increased from 25% to 45–46%. At 30 min, the effect is smaller, but still significant (70% enhancement, from 17% to 29%). Thus, these methyl substitutions at the left or the right of the oxoester functionality show that when steric hindrance around the oxoester functionality is increased, the stability of the oxoester in the plasma is increased as well.

### 3.3. In Vitro Inhibitory Potency and Selectivity of Compounds GK587 and GK639

**Figure 4:**
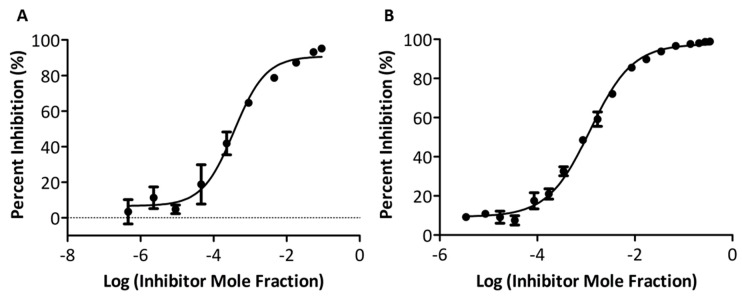
Dose-response inhibition curves for GIVA cPLA_2_ inhibitors GK587 (**A**) and GK639 (**B**). The curves were generated using GraphPad Prism version 5.04 (GraphPad Software Inc., San Diego CA, USA) with a nonlinear regression targeted at symmetrical sigmoidal curves based on plots of % inhibition vs. log(inhibitor mole fraction). The reported *X*_I_(50) values were calculated from the resultant plots.

Both of the synthesized methyl substituted 2-oxoesters were tested for their in vitro inhibitory activity on recombinant human GIVA cPLA_2_ using a LC/MS lipidomics based mixed micelle assay [25]. In addition, their selectivity over the intracellular human calcium-independent PLA_2_ (GVIA iPLA_2_) as well one secreted PLA_2_ (GV sPLA_2_) was also studied using similar group-specific mixed micelle assays. The inhibition results presented in Table 1 are expressed as both percent inhibition or as *X*_I_(50) values. First, the percent of inhibition for each PLA_2_ enzyme at 0.091 mol fraction of each inhibitor was determined. Then, the *X*_I_(50) values were measured for compounds that displayed greater than 95% inhibition of GIVA cPLA_2_. The *X*_I_(50) is the mole fraction of the inhibitor in the total substrate interface required to inhibit the enzyme activity by 50%. The dose-response inhibition curves for GIVA cPLA_2_ inhibitors GK587 and GK639 are shown in Figure 4.

Both compounds GK587 and GK639 were found to inhibit GIVA cPLA_2_ with *X*_I_(50) values of 0.00036 and 0.0012, respectively (Table 1). It is apparent that the introduction of a methyl group resulted in reduction of the inhibitory potency, when compared with the parent inhibitor GK452, which exhibits a *X*_I_(50) value of 0.000078 [23]. However, GK587 was approximately five times less potent than GK452, while GK639 was approximately fifteen times less potent. These findings indicate that the methyl substitution at either position leads to reduced inhibitory activity, however the substitution on the carbon carrying the ester oxygen causes more potent suppression of the potency. That means the methyl substitution on the α-carbon atom to the oxoester functionality is preferable. Both compounds GK587 and GK639 are selective inhibitors of GIVA cPLA_2_, because both of them did not inhibit GVIA iPLA_2_ and GV sPLA_2_ (Table 1).

The results shown below lead to the conclusion that the methyl substitution on the α-carbon atom to the oxoester functionality is a successful strategy to increase the plasma stability of the oxoester inhibitors, ensuring that the inhibitor retains considerable inhibitory potency.

Synthetic GIVA cPLA_2_ inhibitors are potential novel anti-inflammatory agents. For example, inhibitor GK470, previously developed by our group, was found to suppress the release of arachidonic acid in vitro and to exhibit an anti-inflammatory effect comparable to the reference drug methotrexate, in a prophylactic collagen-induced arthritis model, whereas in a therapeutic model, it showed results comparable to those of the reference drug Enbrel [20]. In addition, synthetic inhibitors may help in clarifying the biological role of GIVA cPLA_2_ and the inter-connection with other enzymes. As an example, the involvement of both PLA_2_ and phospholipase D (PLD) in the signaling through phosphatidic acid seems important for cancer cell survival [30,31].

## 4. Conclusions

In conclusion, in the present work we designed and synthesized two new 2-oxoester compounds in an effort to increase their metabolic stability. Determining their in vitro plasma stability and their in vitro inhibitory activity on GIVA cPLA_2_, led us to conclude that inhibitor GK587, in which a methyl group was introduced on the α-carbon atom to the oxoester functionality, exhibits increased metabolic stability retaining at the same time considerable inhibitory potency. Thus, including an α-methyl substitution is a promising way of improving the pharmacological properties of 2-oxoester inhibitors and suggests that further chemical modification of this inhibitor class has the potential for pharmaceutical development of potent, metabolically stable, and selective inhibitors of phospholipase A_2_.

## Figures and Tables

**Table 1:** In vitro Potency and Selectivity of Methyl Substituted 2-Oxoesters.

Compound	Structure	GIVA cPLA_2_		GIVA iPLA_2_	GIVA sPLA_2_
		% Inhibition^a^	*X*_I_(50)	% Inhibition^a^	% Inhibition^a^
**GK452**	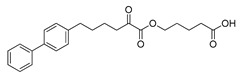	>95%	0.000078 ± 0.00001^b^	N.D.^b,c^	N.D.^b,c^
**GK587**	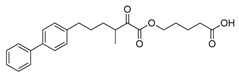	>95%	0.00036 ± 0.00007	N.D.^c^	N.D.^c^
**GK639**	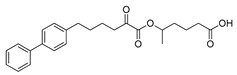	>95%	0.0012 ± 0.00008	N.D.^c^	N.D.^c^

^a^ % Inhibition at 0.091 mole fraction of each inhibitor; ^b^ data taken from ref. [23]. ^c^ N.D. signifies compounds with less than 25% inhibition (or no detectable inhibition).

## Supplementary Materials

HRMS and plasma stability studies as well as copies of the ^1^H and ^13^C NMR spectra are available online at https://www.mdpi.com/2218-273X/10/3/491/s1.
