# Supplementary material for: 2-Oxoester Phospholipase A2 Inhibitors with Enhanced Metabolic Stability
Source: Biomolecules. 2020 Mar 24;10(3):491. doi: 10.3390/biom10030491 (PMC7175278; doi:10.3390/biom10030491)
Supplement: Supplementary file 1 [file biomolecules-10-00491-s001.pdf]

2-Oxoester Phospholipase A_2 Inhibitors With Enhanced Metabolic Stability

Giorgos S. Koutoulogenis,[1] Maroula G. Kokotou,[1] Daiki Hayashi,[2] Varnavas D. Mouchlis,[2] Edward A. Dennis,[2,*] George Kokotos[1,*]

[1]Department of Chemistry, National and Kapodistrian University of Athens, Panepistimiopolis, Athens 15771, Greece

[2]Department of Chemistry and Biochemistry and Department of Pharmacology, School of Medicine, University of California, San Diego, La Jolla, California 92093-0601, USA

SUPPLEMENTARY MATERIALS

	**Page**
**HRMS and Plasma Stability Studies**	**2**
**NMR Spectra**	**4**

Plasma Stability of 2-Oxoester Inhibitors

The *in vitro* stabilities of the 2-oxoester inhibitors were studied in human plasma. The reactions were initiated by the addition of test compound to a 250 μL of preheated plasma solution to yield a final concertation of 1 mg/L. Samples (50 μL) were obtained at 0, 15, 30, 60 and 90 min and acetonitrile (200 μL) was added. The samples were subjected to vortex mixing for 1 min and then centrifugation for 10 min. The clear supernatants were analyzed by LC-HRMS/MS.

As an example, the full scan ESI-HRMS spectrum of inhibitor GK639 is depicted in Figure 1. The chemical formulas of the parent and the fragment ions were also identified using the Formula Finder tab from Peak View 2.1. The MS Rank values, as well as the RDB values, for the elemental compositions of the two ions are presented in Figure 1. The MS Rank order is based on the MS data using a combination of mass accuracy and match to the theoretical isotope pattern. The RDB value is a formal calculation of the sum of the number of rings and double bonds present in the formula.

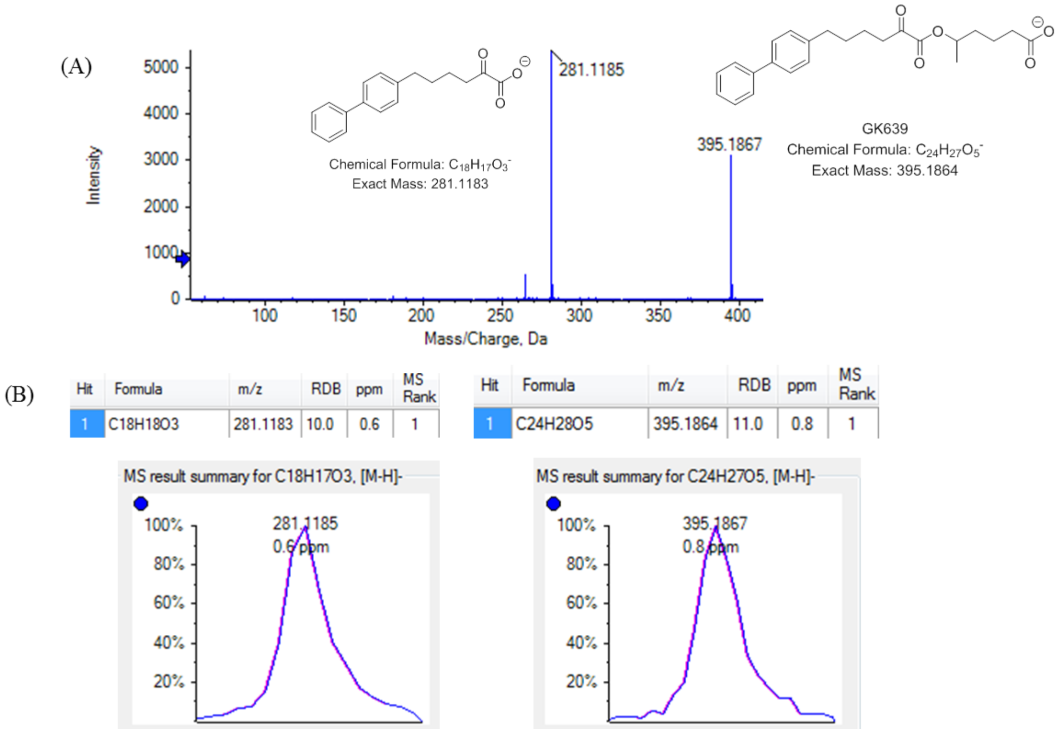

Supporting Figure 1. Full scan ESI-HRMS spectrum of GK639 (A) and mass spectral data for the parent and the fragment ions (B).

The extracted ion chromatograms (EICs) of a standard GK639 sample (1 mg/L) and plasma samples that contain inhibitor GK639, taken at 15, 30, 60, 90 min, are shown in Figure 2.

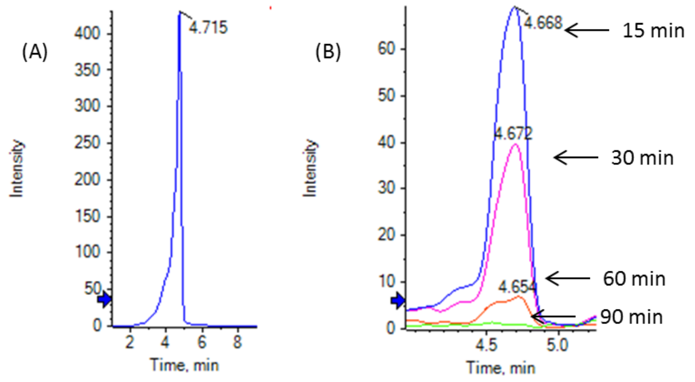

Supporting Figure 2. Extracted ion chromatograms (EICs) of a standard GK639 sample (1 mg/L) (A) and plasma samples that contain GK639, taken at 15, 30, 60, 90 min (B).

NMR Spectra

(4)

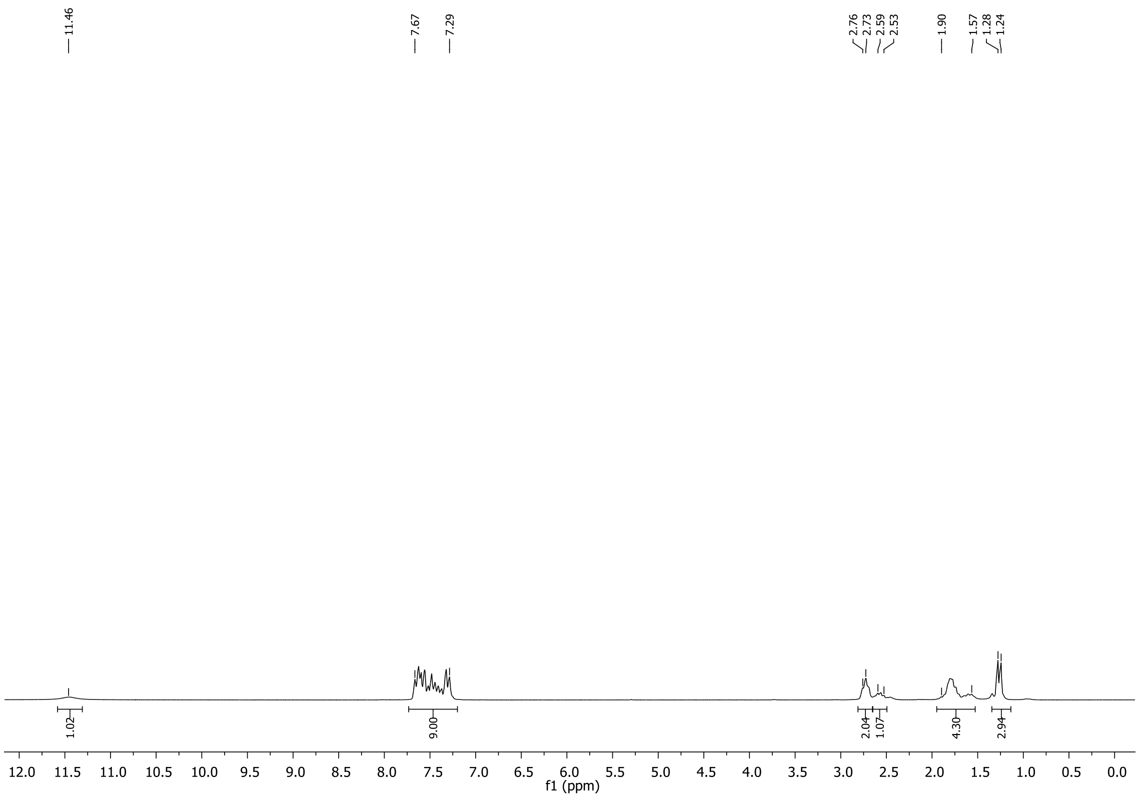

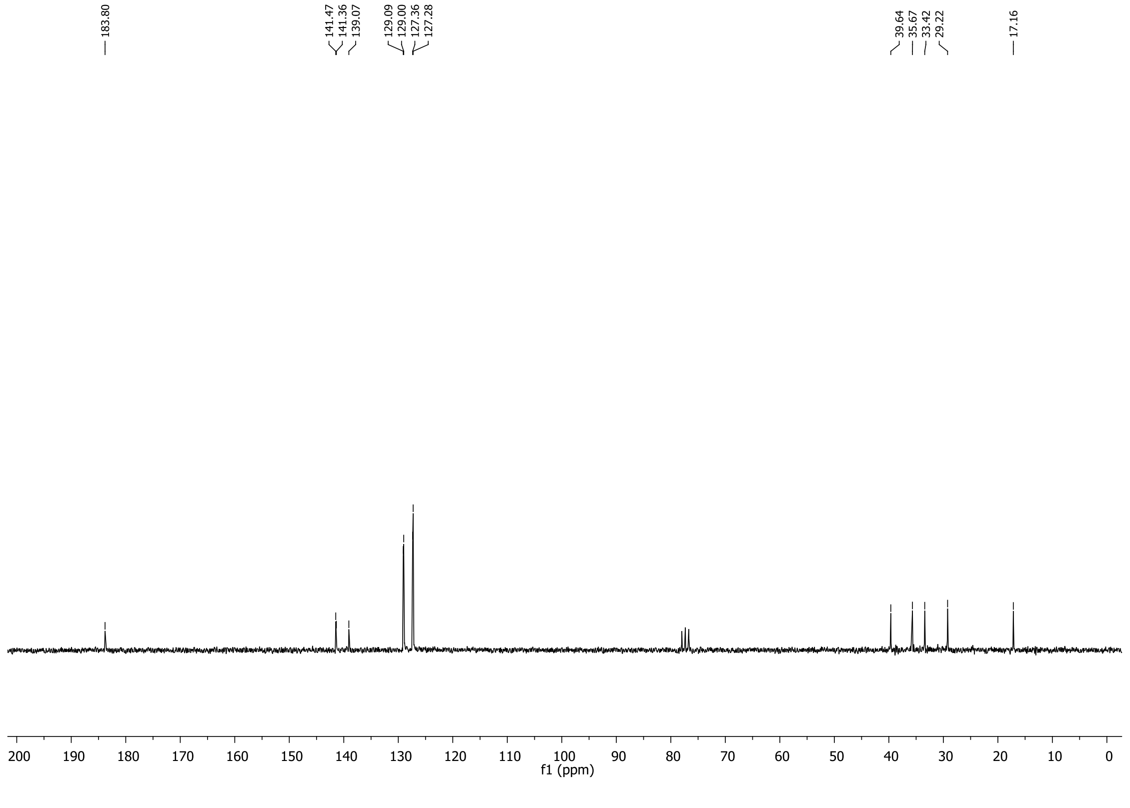

(5)

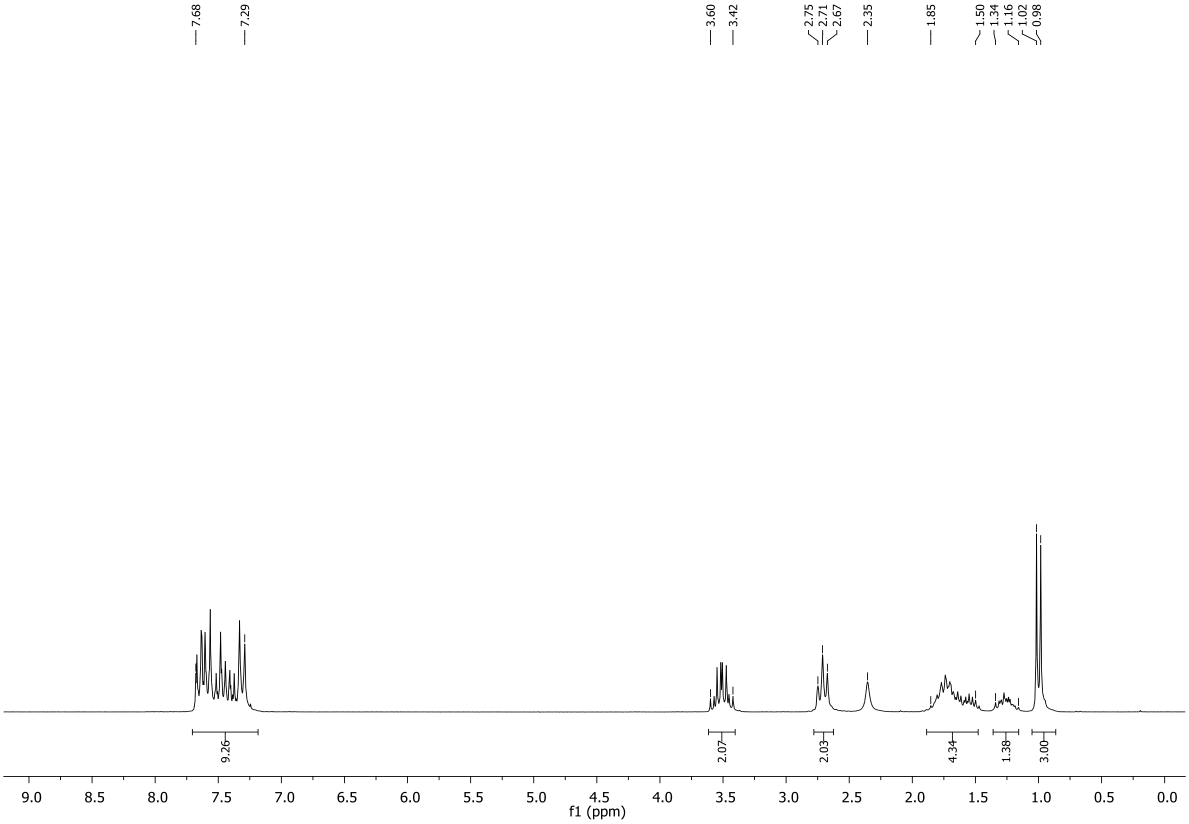
7.68
7.29
3.60
3.42
2.75
2.71
2.67
2.35
1.85
1.50
1.34
1.16
1.02
0.98
9.26
2.07
2.03
4.34
1.38
3.00
9.0
8.5
8.0
7.5
7.0
6.5
6.0
5.5
5.0
4.5
4.0
3.5
3.0
2.5
2.0
1.5
1.0
0.5
0.0
f1 (ppm)

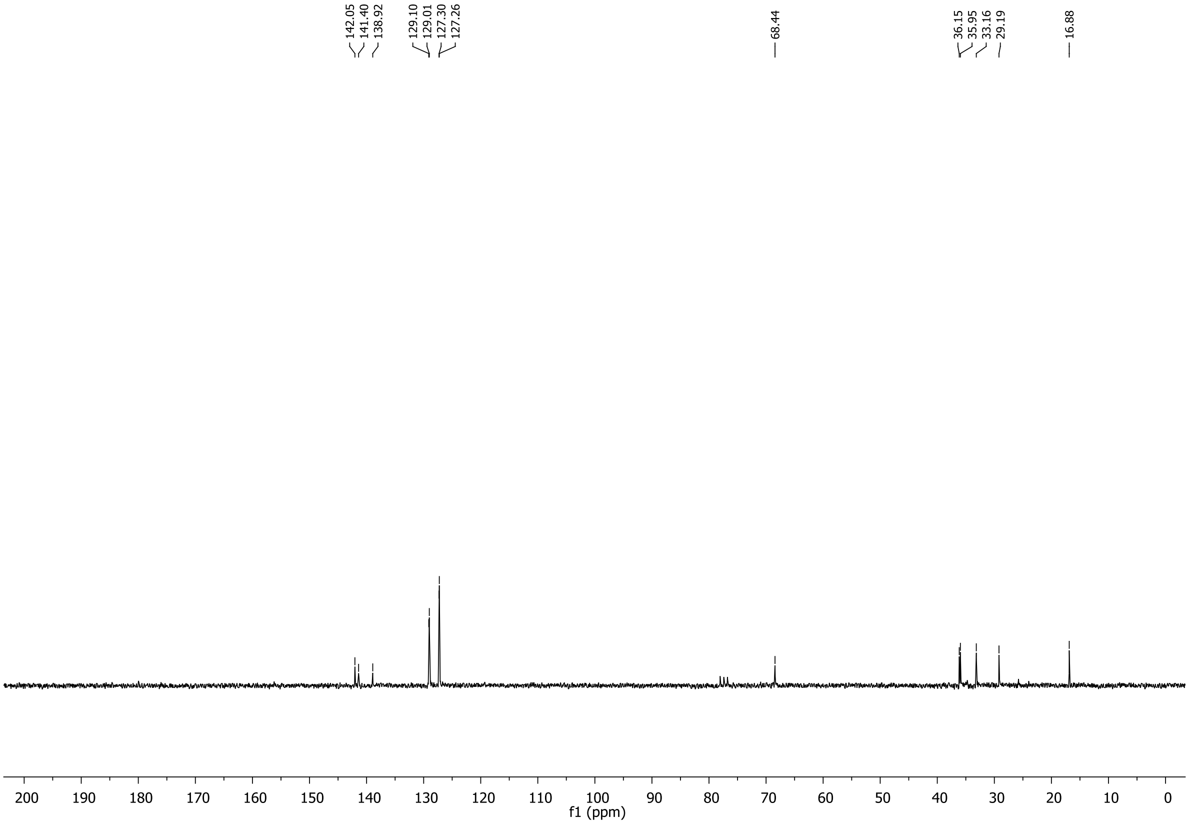
142.05
141.40
138.92
129.10
129.01
127.30
127.26
68.44
36.15
35.95
33.16
29.19
16.88
200
190
180
170
160
150
140
130
120
110
100
90
80
70
60
50
40
30
20
10
0
f1 (ppm)

(7)

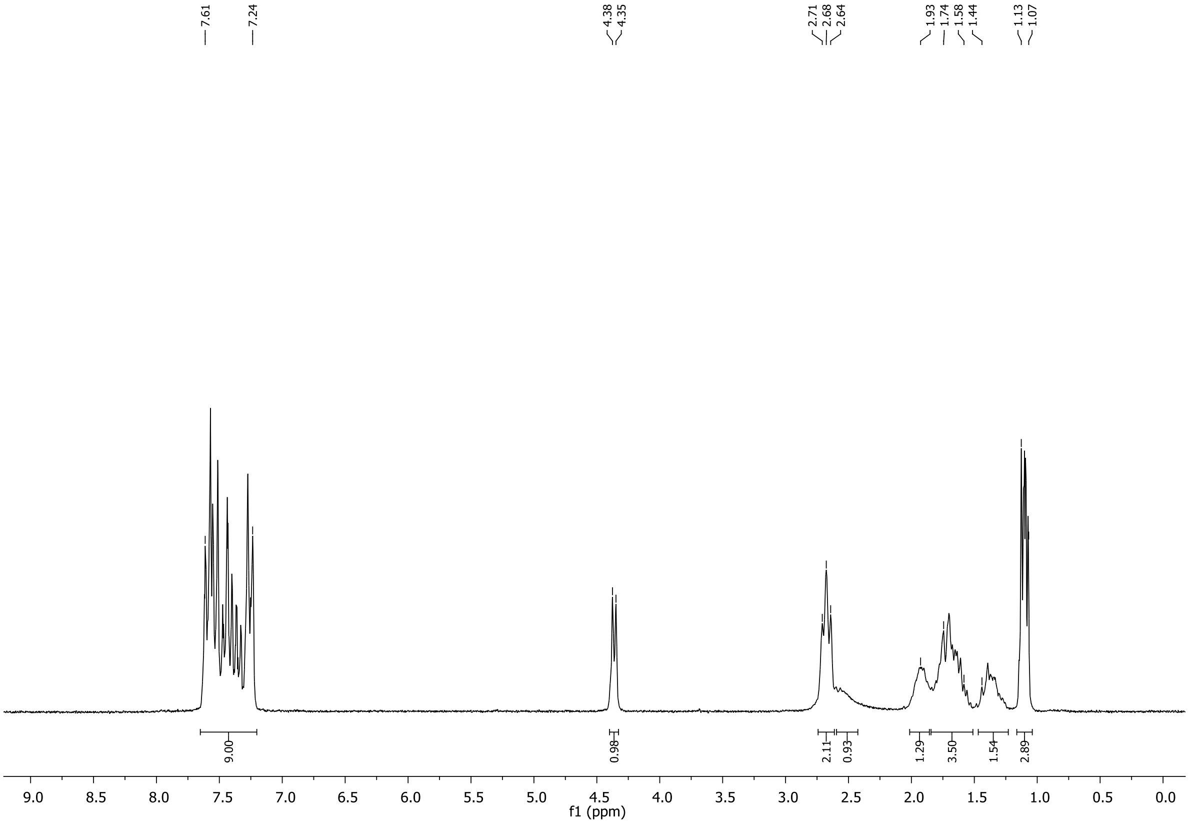

7.61
7.24
4.38
4.35
2.71
2.68
2.64
1.93
1.74
1.58
1.44
1.13
1.07
9.00
0.98
2.11
0.93
1.29
3.50
1.54
2.89
9.0 8.5 8.0 7.5 7.0 6.5 6.0 5.5 5.0 4.5 4.0 3.5 3.0 2.5 2.0 1.5 1.0 0.5 0.0
f1 (ppm)

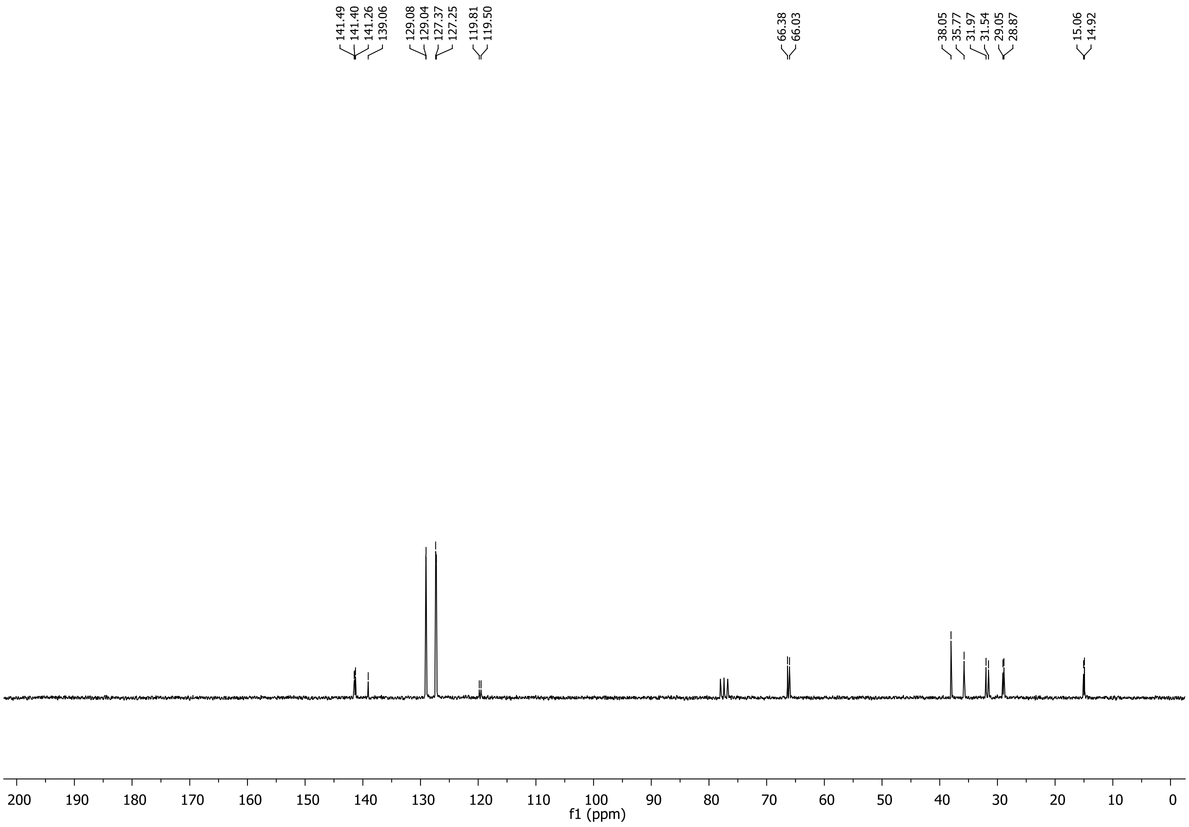

141.49
141.40
141.26
139.06
129.08
129.04
127.37
127.25
119.81
119.50
66.38
66.03
38.05
35.77
31.97
31.54
29.05
28.87
15.06
14.92
200 190 180 170 160 150 140 130 120 110 100 90 80 70 60 50 40 30 20 10 0
f1 (ppm)

(8)

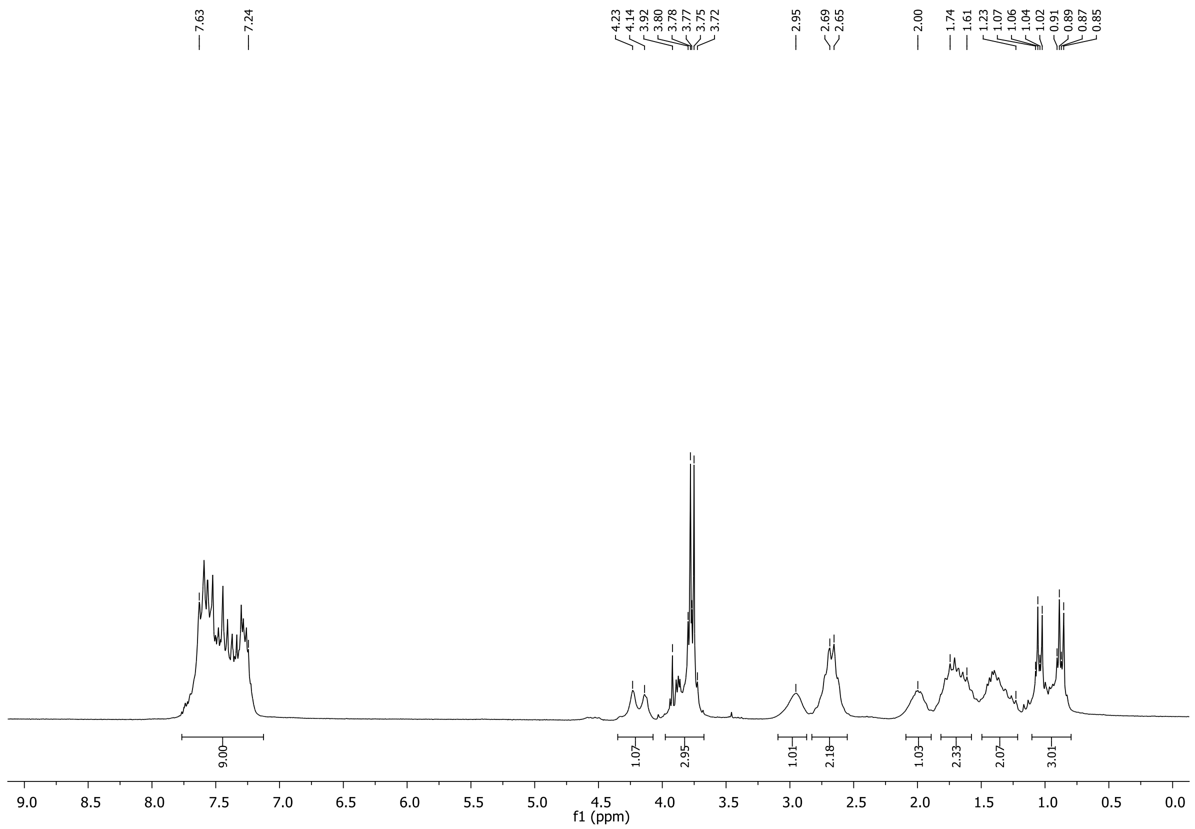
7.63
7.24
4.23
4.14
3.92
3.80
3.78
3.77
3.75
3.72
2.95
2.69
2.65
2.00
1.74
1.61
1.23
1.07
1.06
1.04
1.02
0.91
0.89
0.87
0.85
9.00
1.07
2.95
1.01
2.18
1.03
2.33
2.07
3.01
9.0 8.5 8.0 7.5 7.0 6.5 6.0 5.5 5.0 4.5 4.0 3.5 3.0 2.5 2.0 1.5 1.0 0.5 0.0
f1 (ppm)

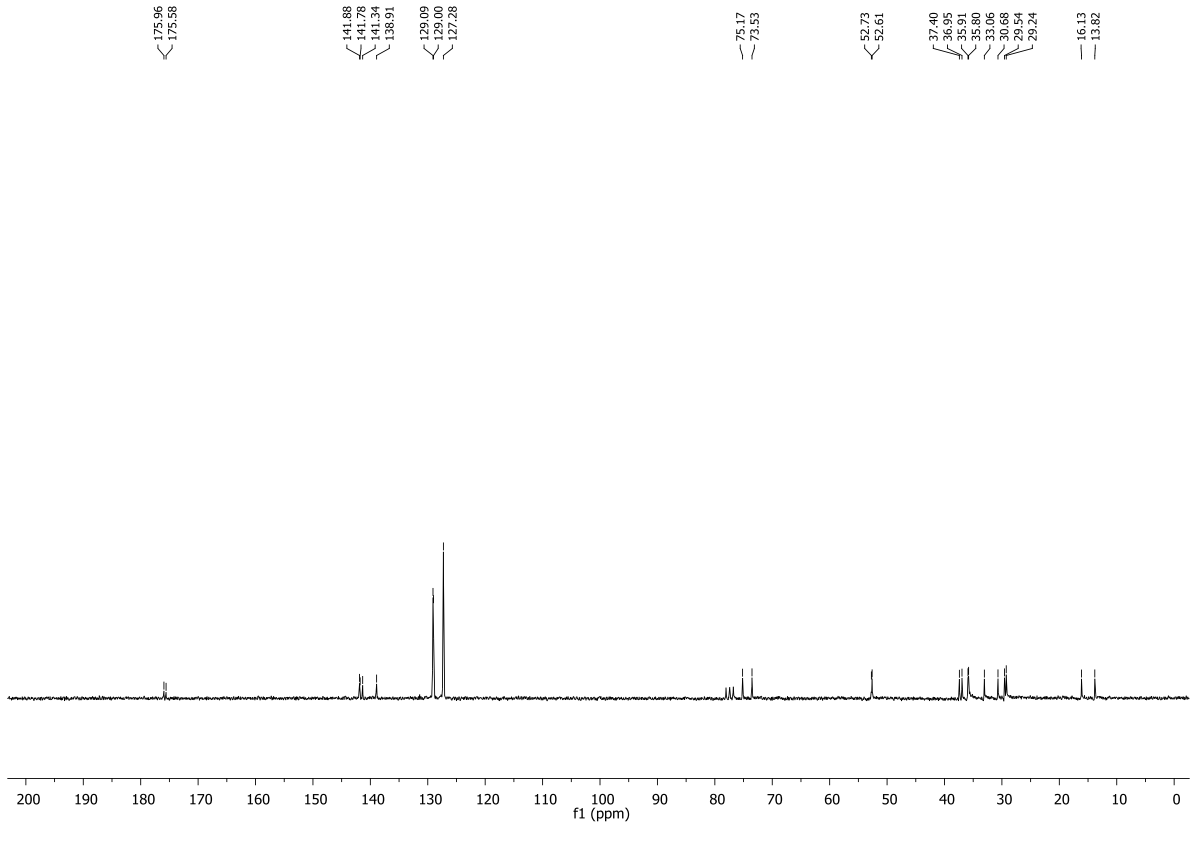
175.96
175.58
141.88
141.78
141.34
138.91
129.09
129.00
127.28
75.17
73.53
52.73
52.61
37.40
36.95
35.91
35.80
33.06
30.68
29.54
29.24
16.13
13.82
200 190 180 170 160 150 140 130 120 110 100 90 80 70 60 50 40 30 20 10 0
f1 (ppm)

(9)

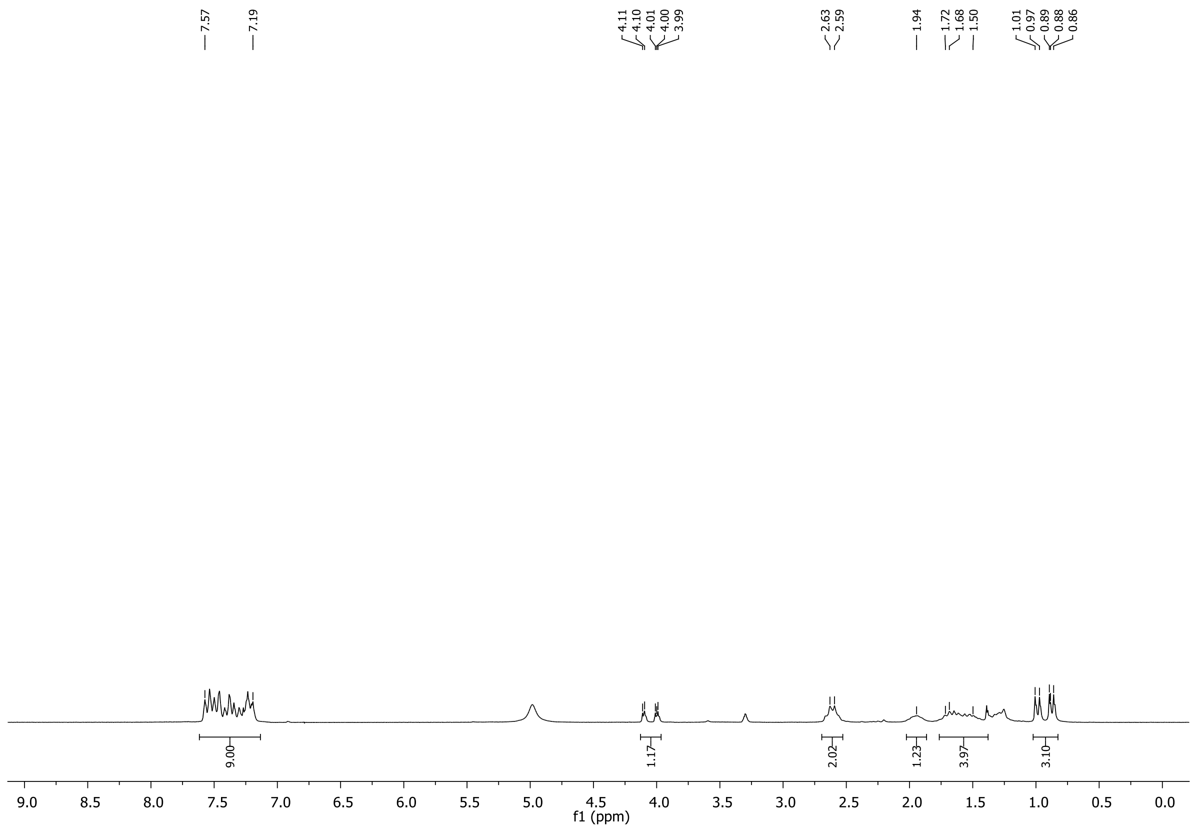
7.57
7.19
4.11
4.10
4.01
4.00
3.99
2.63
2.59
1.94
1.72
1.68
1.50
1.01
0.97
0.89
0.88
0.86
9.00
1.17
2.02
1.23
3.97
3.10
f1 (ppm)

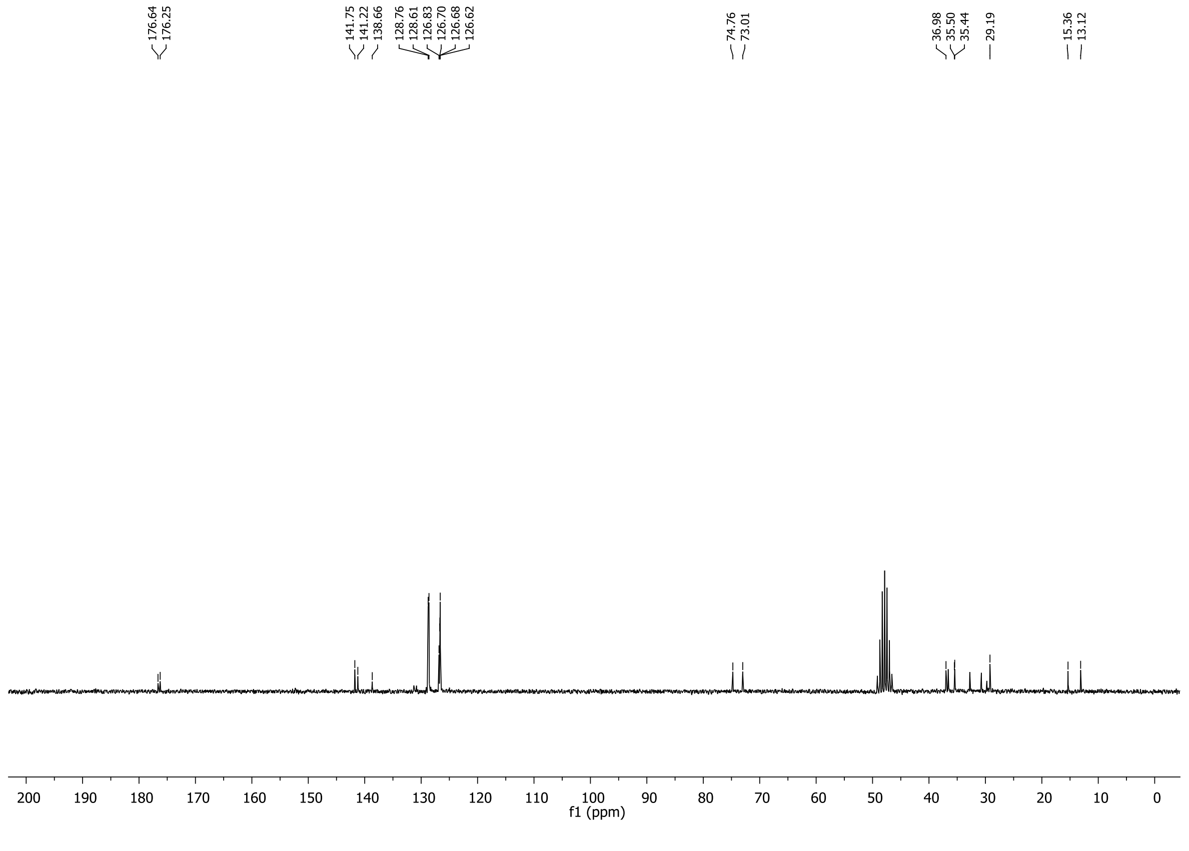
176.64
176.25
141.75
141.22
138.66
128.76
128.61
126.83
126.70
126.68
126.62
74.76
73.01
36.98
35.50
35.44
29.19
15.36
13.12
f1 (ppm)

(12)

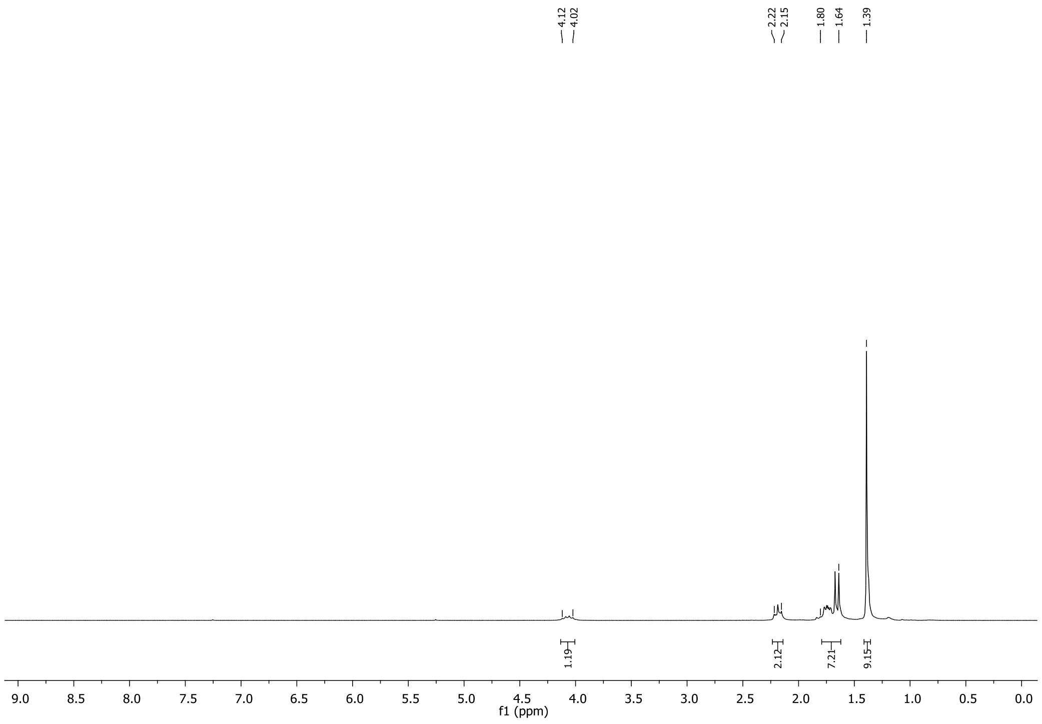
4.12
4.02
2.22
2.15
1.80
1.64
1.39
1.19
2.12
7.21
9.15
9.0 8.5 8.0 7.5 7.0 6.5 6.0 5.5 5.0 4.5 4.0 3.5 3.0 2.5 2.0 1.5 1.0 0.5 0.0
f1 (ppm)

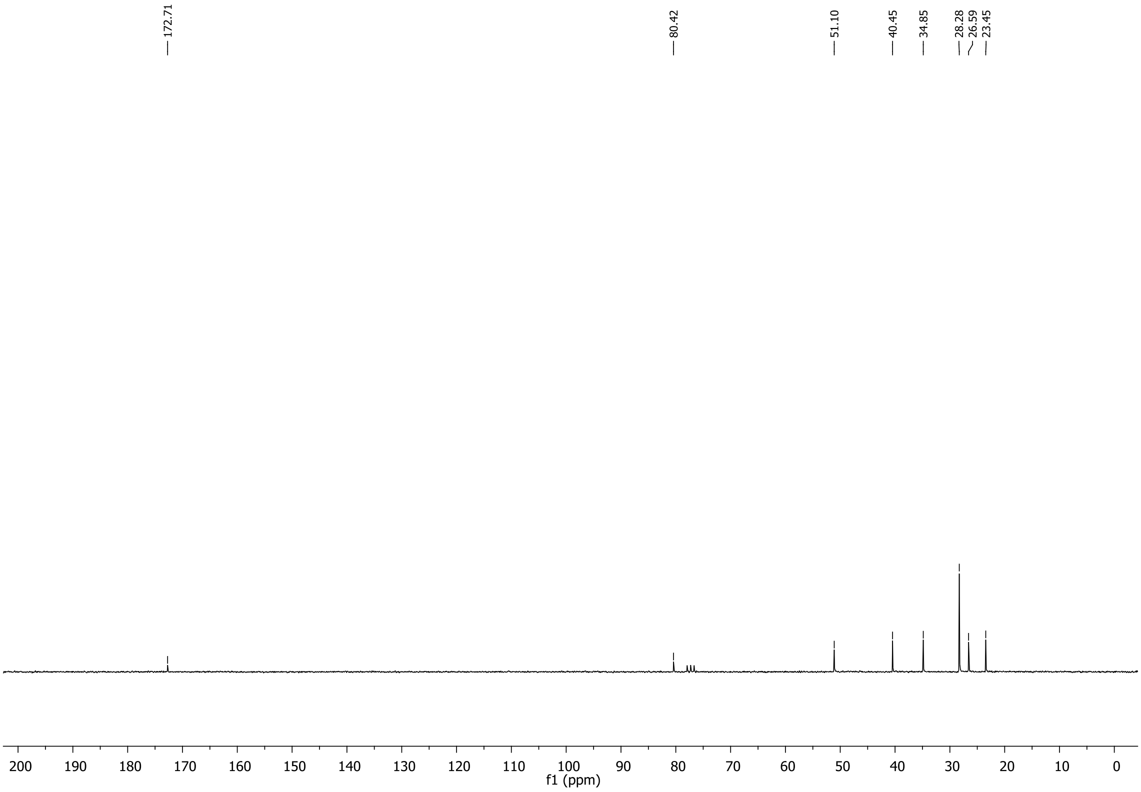
172.71
80.42
51.10
40.45
34.85
28.28
26.59
23.45
200 190 180 170 160 150 140 130 120 110 100 90 80 70 60 50 40 30 20 10 0
f1 (ppm)

(15a)

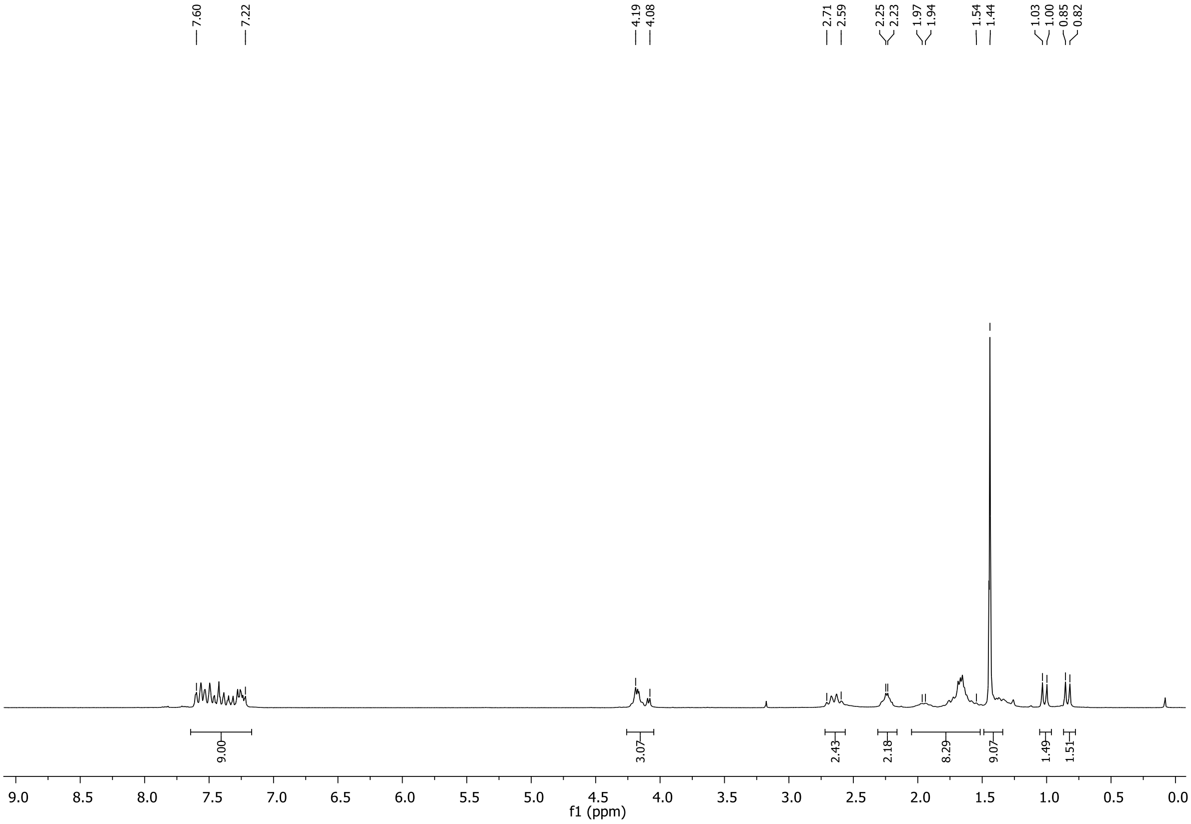
7.60
7.22
4.19
4.08
2.71
2.59
2.25
2.23
1.97
1.94
1.54
1.44
1.03
1.00
0.85
0.82
9.00
3.07
2.43
2.18
8.29
9.07
1.49
1.51
f1 (ppm)

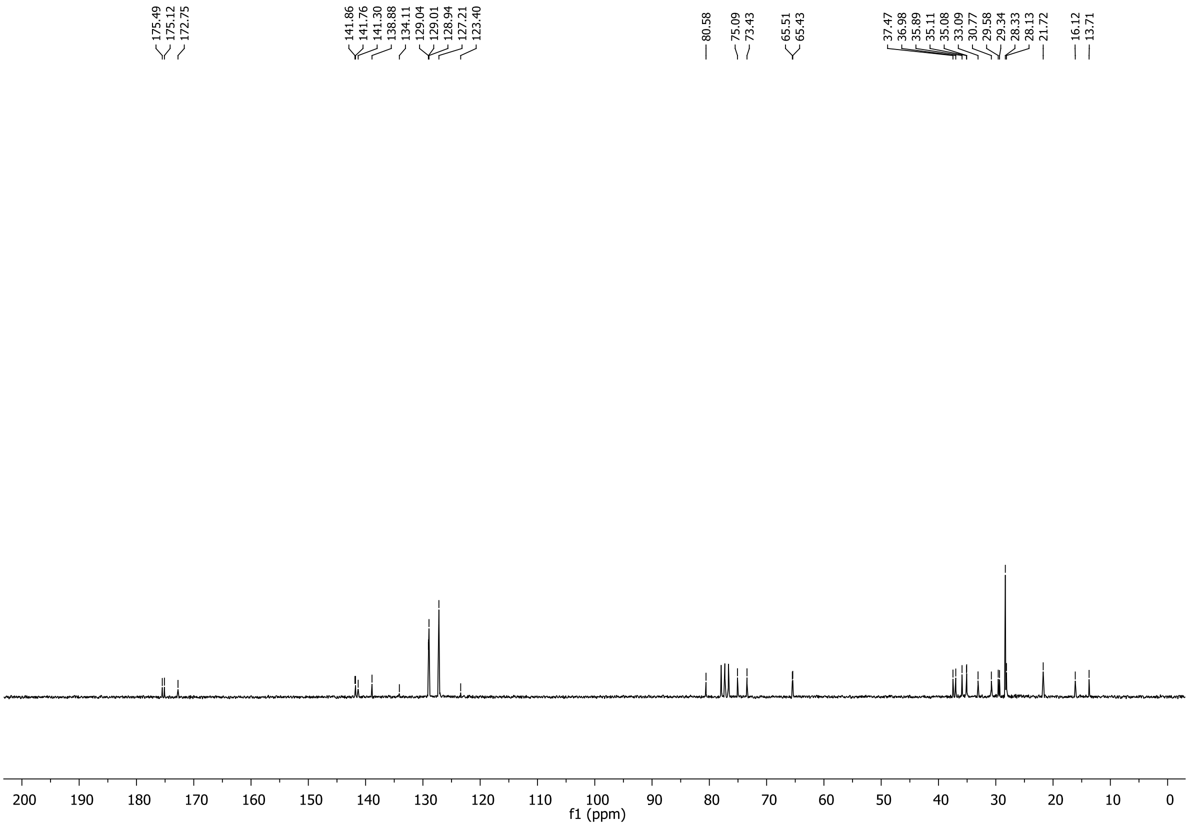
175.49
175.12
172.75
141.86
141.76
141.30
138.88
134.11
129.04
129.01
128.94
127.21
123.40
80.58
75.09
73.43
65.51
65.43
37.47
36.98
35.89
35.11
35.08
33.09
30.77
29.58
29.34
28.33
28.13
21.72
16.12
13.71
f1 (ppm)

(15b)

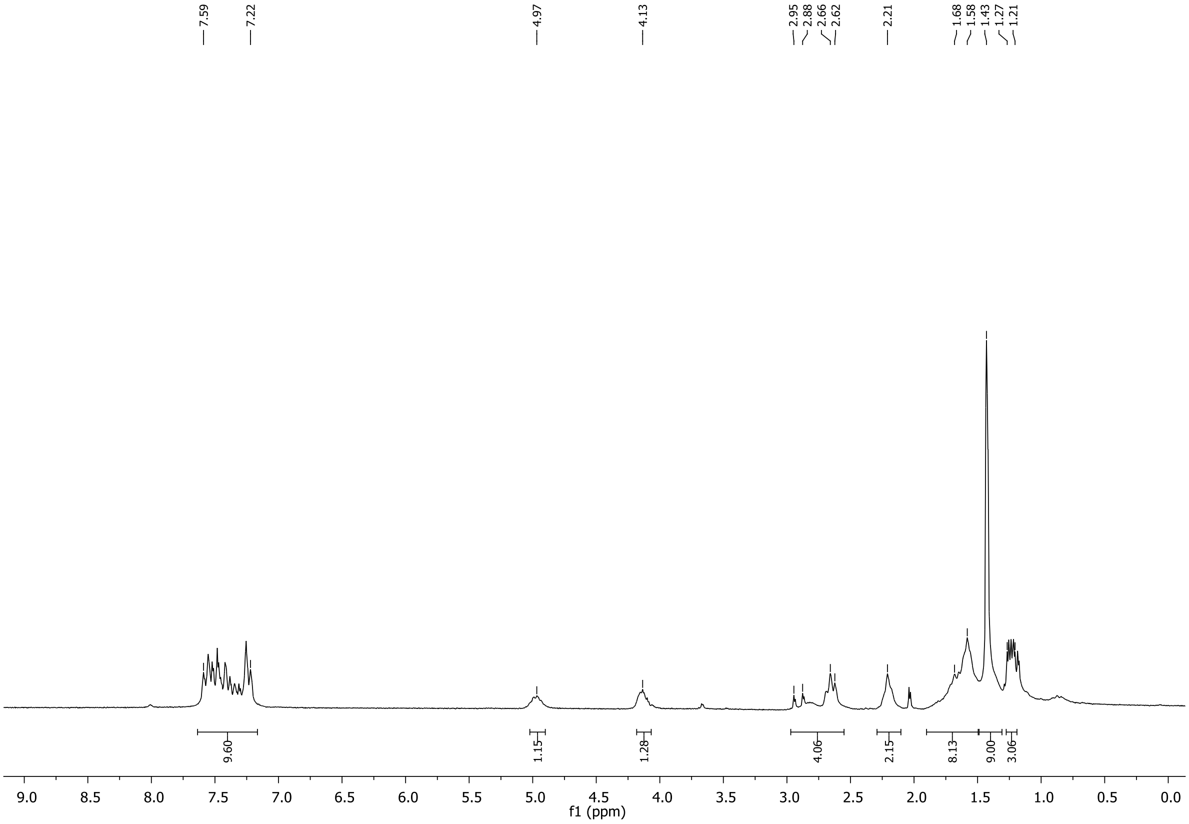
7.59
7.22
4.97
4.13
2.95
2.88
2.66
2.62
2.21
1.68
1.58
1.43
1.27
1.21
9.60
1.15
1.28
4.06
2.15
8.13
9.00
3.06
9.0 8.5 8.0 7.5 7.0 6.5 6.0 5.5 5.0 4.5 4.0 3.5 3.0 2.5 2.0 1.5 1.0 0.5 0.0
f1 (ppm)

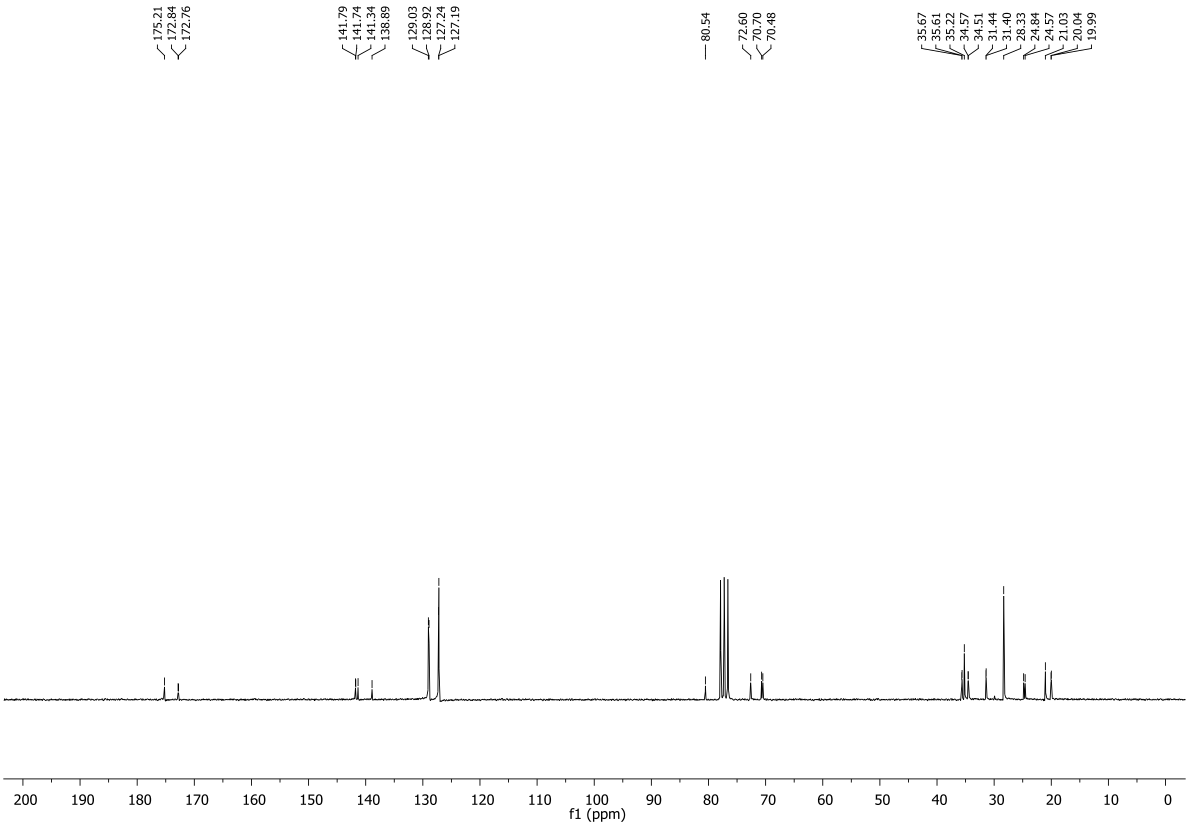
175.21
172.84
172.76
141.79
141.74
141.34
138.89
129.03
128.92
127.24
127.19
80.54
72.60
70.70
70.48
35.67
35.61
35.22
34.57
34.51
31.44
31.40
28.33
24.84
24.57
21.03
20.04
19.99
200 190 180 170 160 150 140 130 120 110 100 90 80 70 60 50 40 30 20 10 0
f1 (ppm)

(16a)

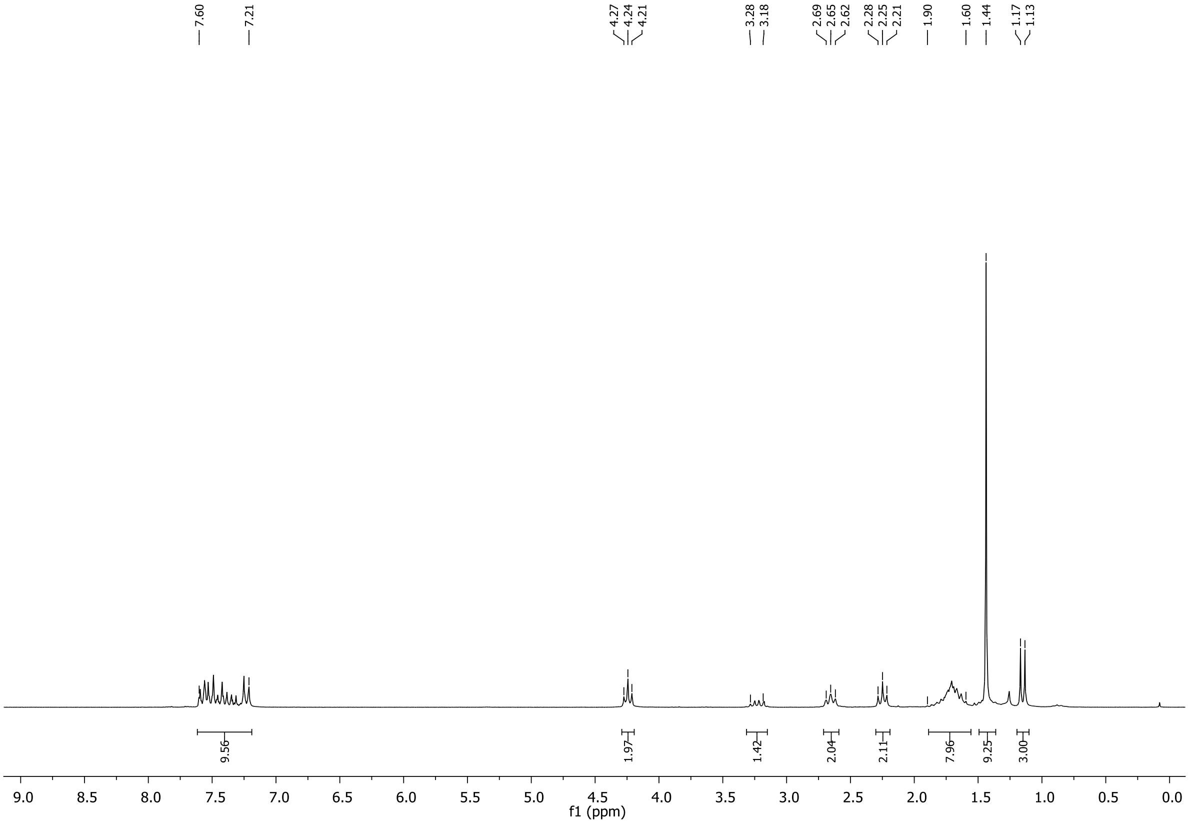
7.60
7.21
4.27
4.24
4.21
3.28
3.18
2.69
2.65
2.62
2.28
2.25
2.21
1.90
1.60
1.44
1.17
1.13
9.56
1.97
1.42
2.04
2.11
7.96
9.25
3.00
f1 (ppm)

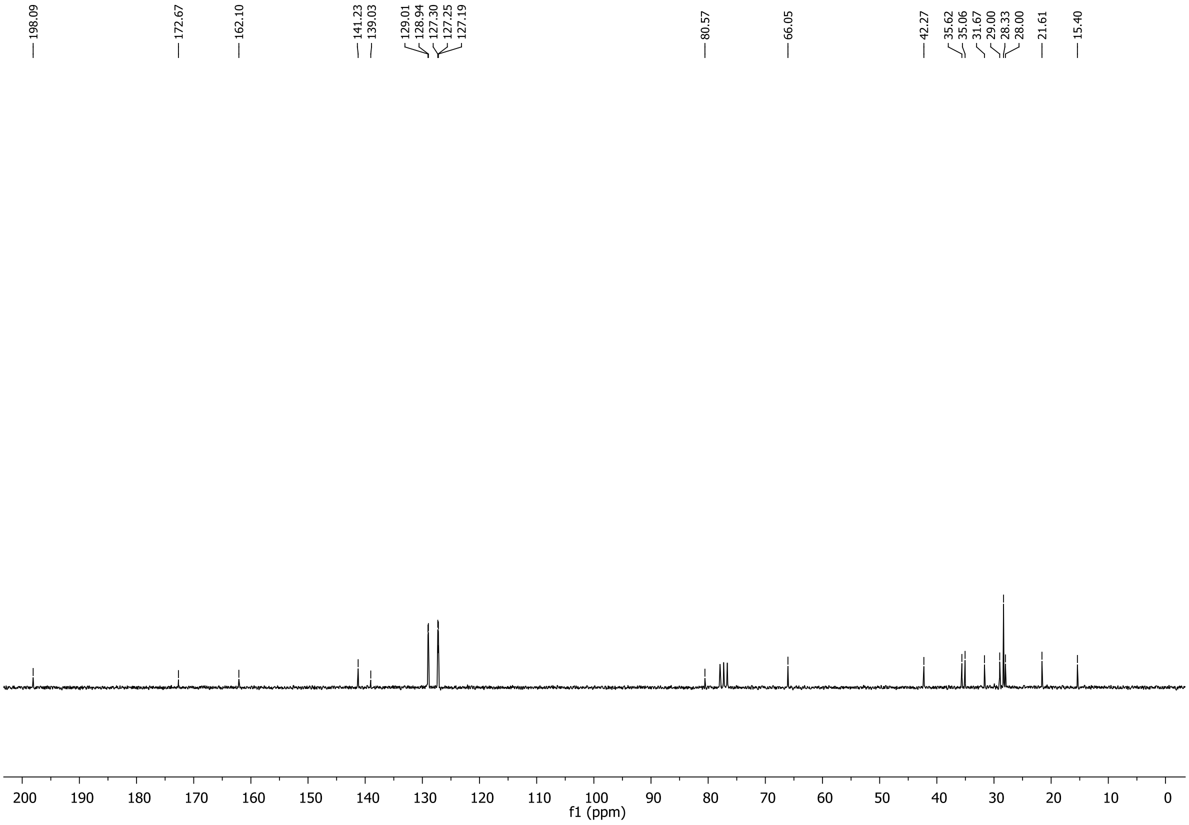
198.09
172.67
162.10
141.23
139.03
129.01
128.94
127.30
127.25
127.19
80.57
66.05
42.27
35.62
35.06
31.67
29.00
28.33
28.00
21.61
15.40
f1 (ppm)

(16b)

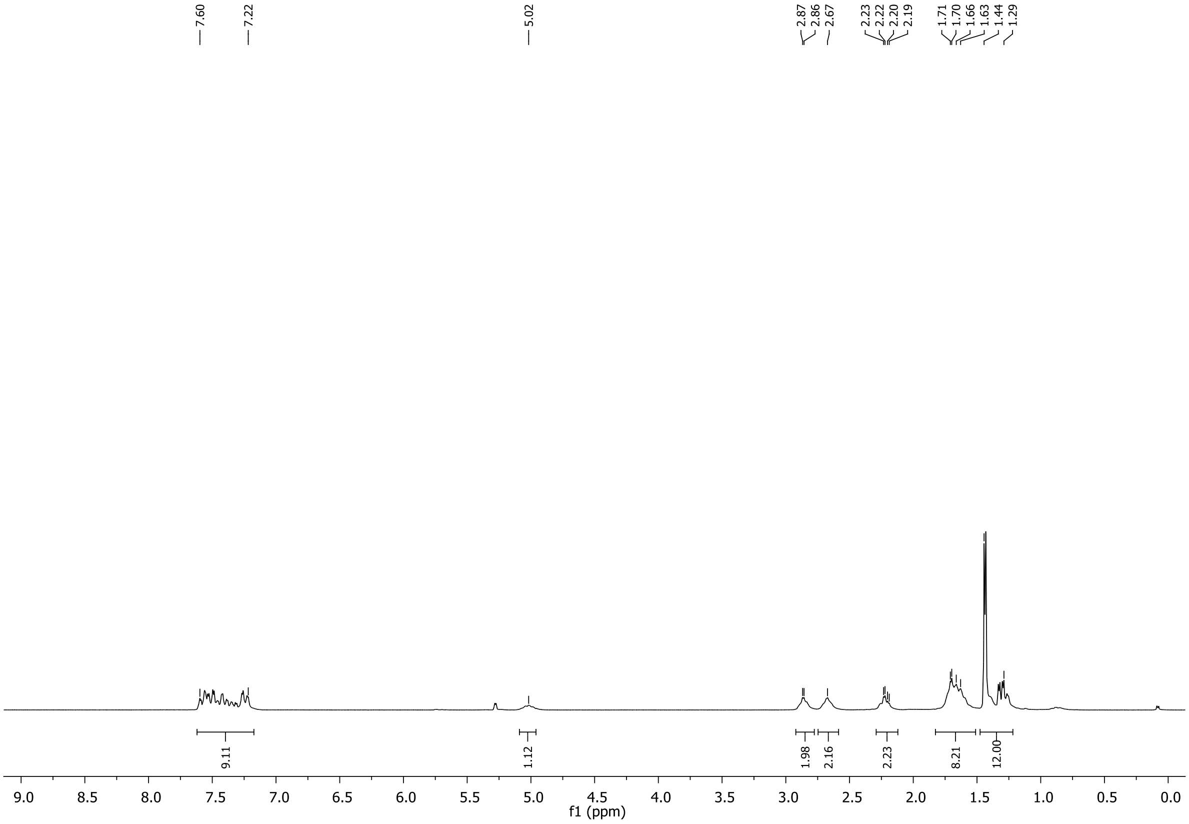

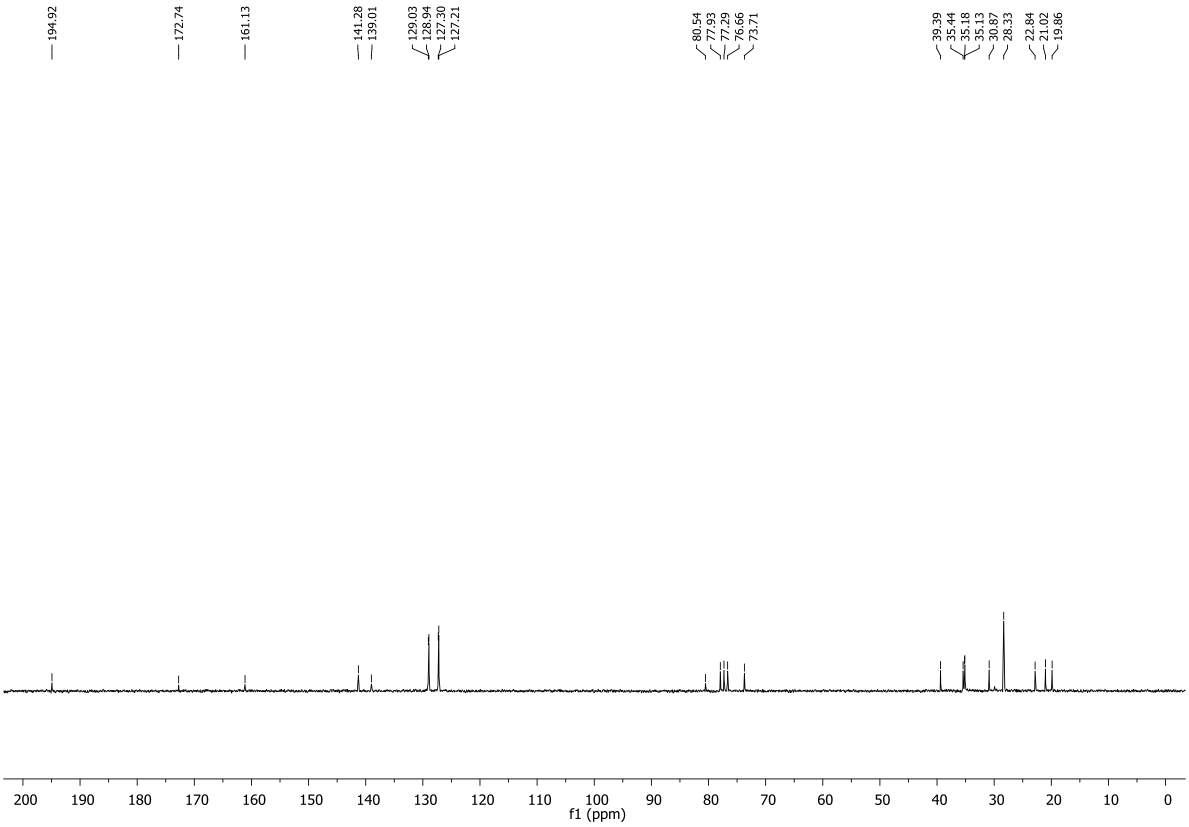

(17a)

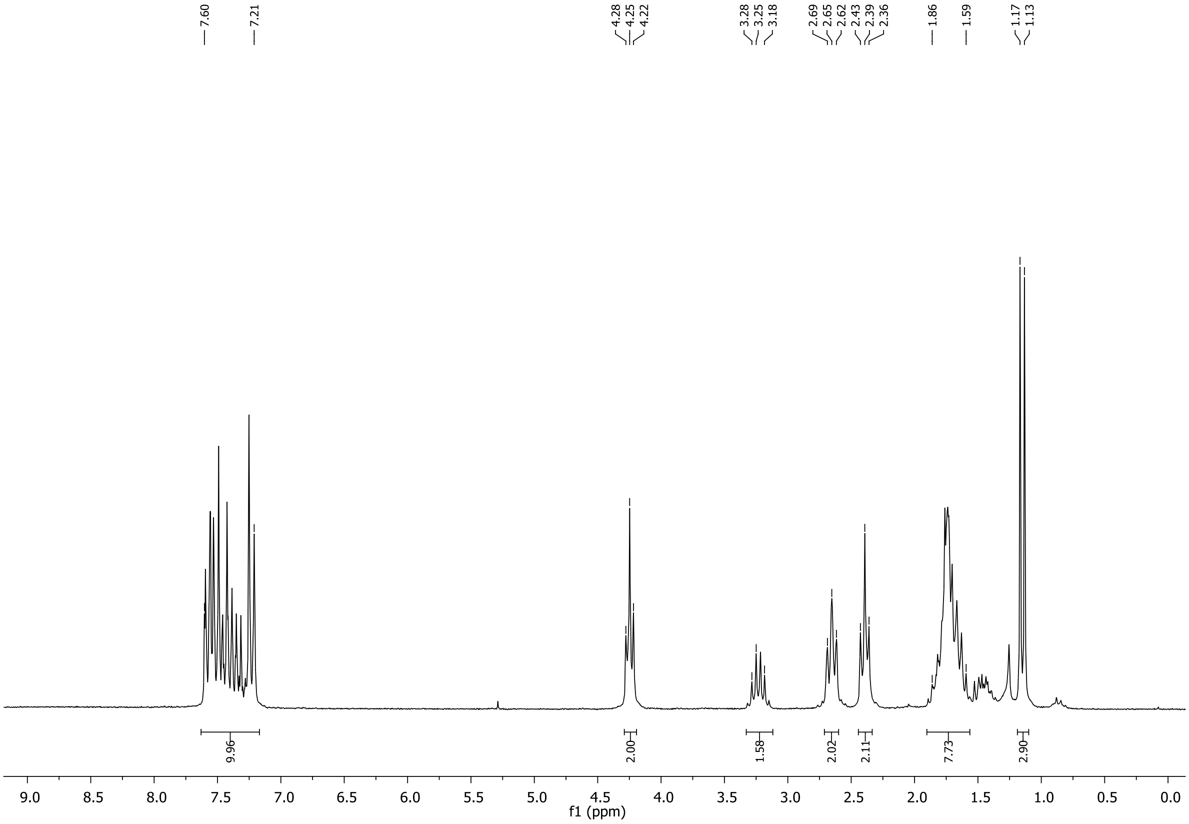
7.60
7.21
4.28
4.25
4.22
3.28
3.25
3.18
2.69
2.65
2.62
2.43
2.39
2.36
1.86
1.59
1.17
1.13
9.96
2.00
1.58
2.02
2.11
7.73
2.90
f1 (ppm)

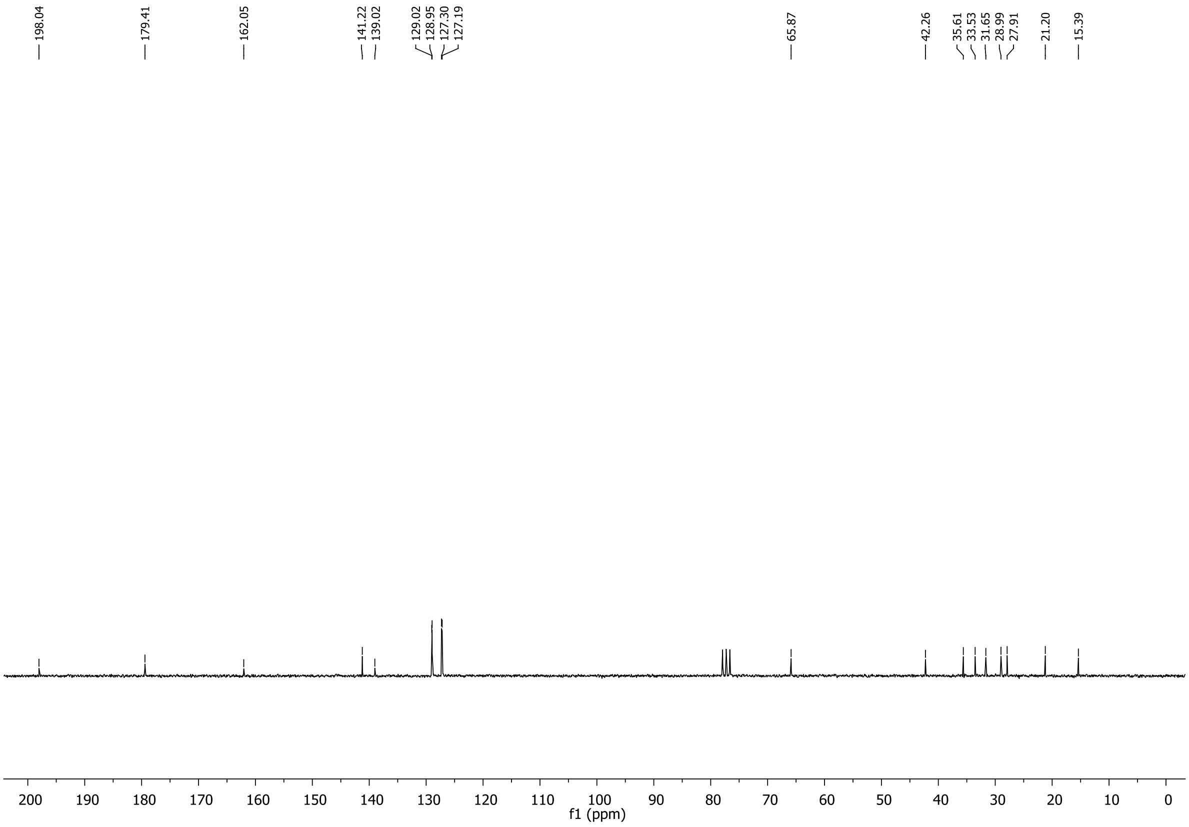
198.04
179.41
162.05
141.22
139.02
129.02
128.95
127.30
127.19
65.87
42.26
35.61
33.53
31.65
28.99
27.91
21.20
15.39
f1 (ppm)

(17b)

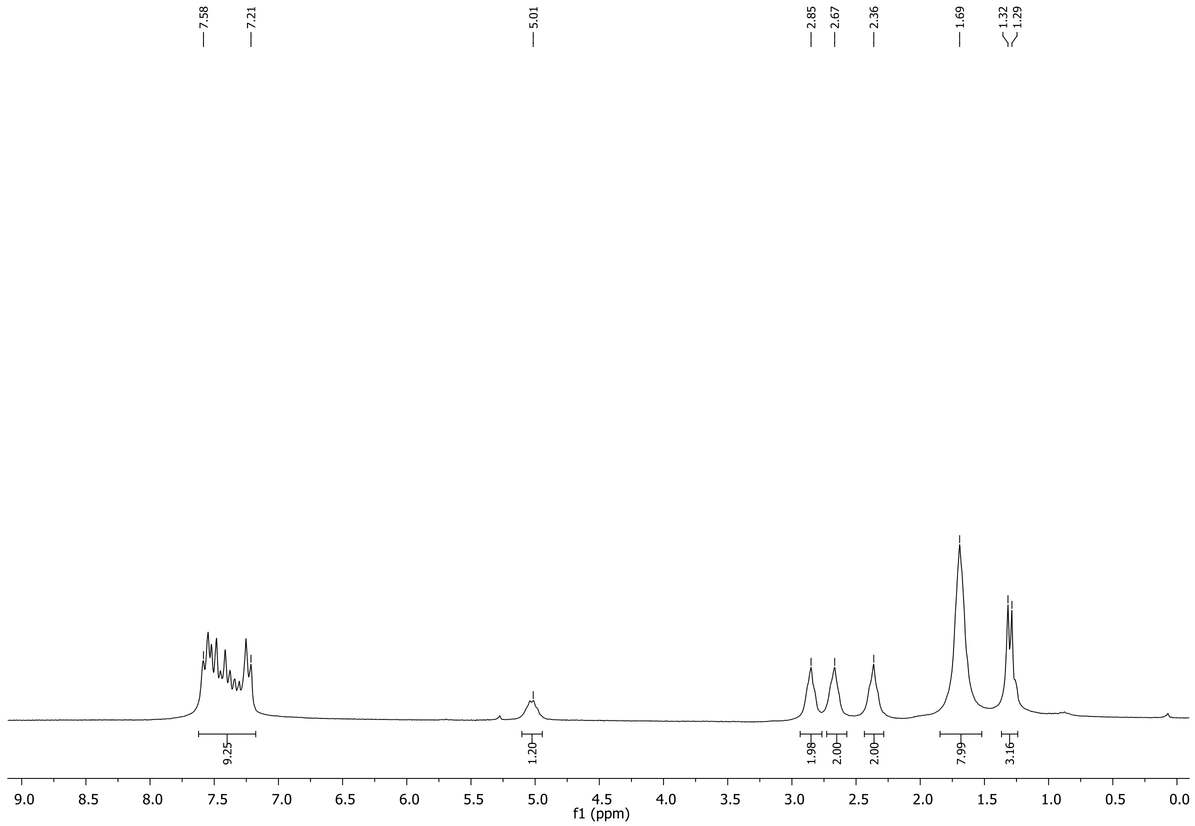
7.58
7.21
5.01
2.85
2.67
2.36
1.69
1.32
1.29
9.25
1.20
1.98
2.00
2.00
7.99
3.16
9.0 8.5 8.0 7.5 7.0 6.5 6.0 5.5 5.0 4.5 4.0 3.5 3.0 2.5 2.0 1.5 1.0 0.5 0.0
f1 (ppm)

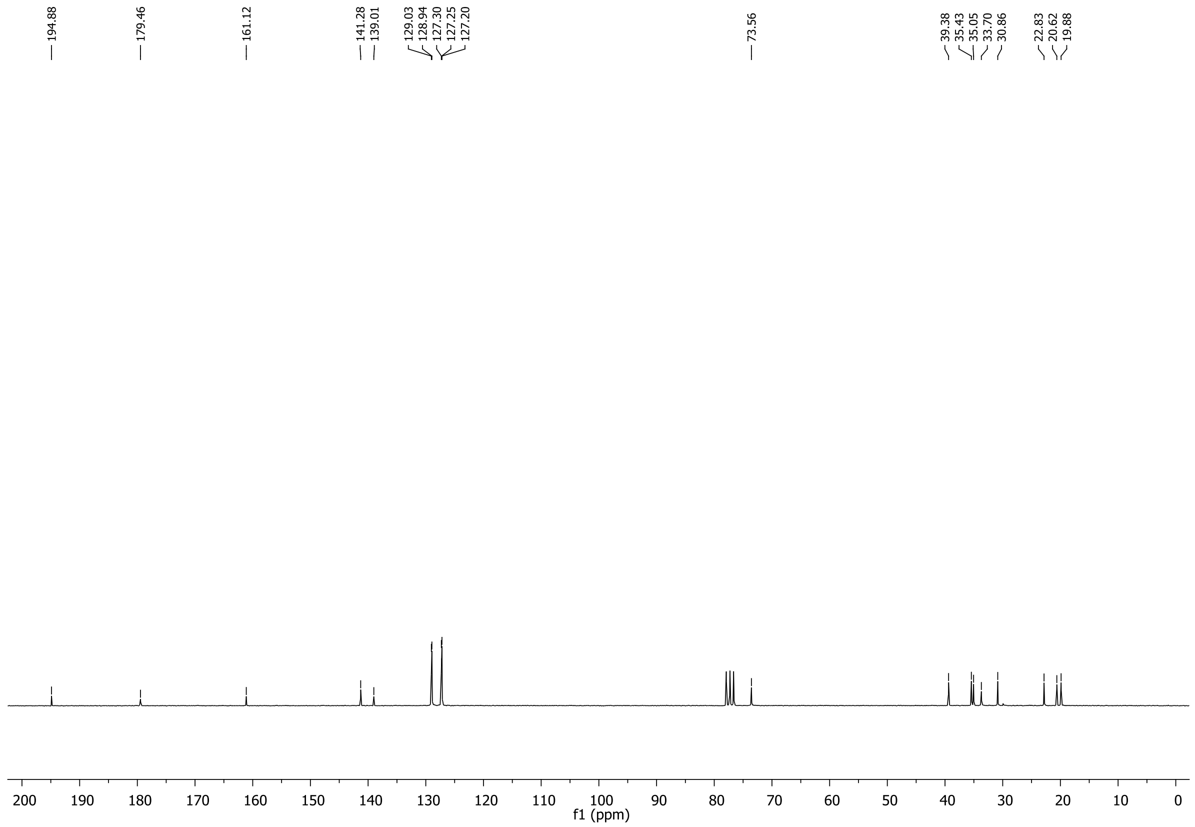
194.88
179.46
161.12
141.28
139.01
129.03
128.94
127.30
127.25
127.20
73.56
39.38
35.43
35.05
33.70
30.86
22.83
20.62
19.88
200 190 180 170 160 150 140 130 120 110 100 90 80 70 60 50 40 30 20 10 0
f1 (ppm)
